# Ancestry of the AUTS2 family–A novel group of polycomb-complex proteins involved in human neurological disease

**DOI:** 10.1371/journal.pone.0232101

**Published:** 2020-12-11

**Authors:** Robert A. Sellers, David L. Robertson, May Tassabehji

**Affiliations:** 1 Evolution & Genomic Sciences, School of Biological Sciences, University of Manchester, Manchester, United Kingdom; 2 MRC-University of Glasgow Centre for Virus Research, Garscube Campus, Glasgow, United Kingdom; Monash University - Malaysia Campus, MALAYSIA

## Abstract

Autism susceptibility candidate 2 (*AUTS2*) is a neurodevelopmental regulator associated with an autosomal dominant intellectual disability syndrome, AUTS2 syndrome, and is implicated as an important gene in human-specific evolution. AUTS2 exists as part of a tripartite gene family, the AUTS2 family, which includes two relatively undefined proteins, Fibrosin (FBRS) and Fibrosin-like protein 1 (FBRSL1). Evolutionary ancestors of AUTS2 have not been formally identified outside of the *Animalia* clade. A *Drosophila melanogaster* protein, Tay bridge, with a role in neurodevelopment, has been shown to display limited similarity to the C-terminal of AUTS2, suggesting that evolutionary ancestors of the AUTS2 family may exist within other Protostome lineages. Here we present an evolutionary analysis of the AUTS2 family, which highlights ancestral homologs of AUTS2 in multiple *Protostome* species, implicates AUTS2 as the closest human relative to the progenitor of the AUTS2 family, and demonstrates that Tay bridge is a divergent ortholog of the ancestral *AUTS2* progenitor gene. We also define regions of high relative sequence identity, with potential functional significance, shared by the extended AUTS2 protein family. Using structural predictions coupled with sequence conservation and human variant data from 15,708 individuals, a putative domain structure for AUTS2 was produced that can be used to aid interpretation of the consequences of nucleotide variation on protein structure and function in human disease. To assess the role of *AUTS2* in human-specific evolution, we recalculated allele frequencies at previously identified *human derived* sites using large population genome data, and show a high prevalence of ancestral alleles, suggesting that *AUTS2* may not be a rapidly evolving gene, as previously thought.

## Introduction

Autism susceptibility gene 2 (*AUTS2*) is a neurodevelopmental regulator associated with an autosomal dominant neurological syndrome with ASD-like (Autism Spectrum Disorder-like) features. The AUTS2 syndrome phenotype includes borderline to moderate intellectual disability (ID), microcephaly, feeding difficulties and mild dysmorphic facial features including highly-arched eyebrows, short philtrum, ptosis and micro/retrognathia along with mild abnormalities of the hands and feet **[1]**. Specific ASD-like features, including obsessive or ritualistic behaviours, are frequently displayed, although sociability is largely unaffected **[1]**. Hemizygous deletion of *AUTS2*, alongside deleterious truncating point mutations, have highlighted the C-terminus as being associated with more severe clinical manifestations of the syndrome **[2]**. Homozygous deletion of *Auts2* in mice is prenatally lethal, and heterozygosity results in a similar phenotype to that of patients, including: short stature, reduction in body mass, impaired recognition of learned objects and attenuation of associative memory with no notable social deficit **[3–5]**. Morpholino (MO) knockdown of *Auts2* in zebrafish (*Danio rerio*) results in reduced brain volume and retrognathia, with behavioural abnormalities including slowed swimming speed and a reduced response to tactile stimuli **[6]**. Increased levels of *Auts2* expression have been identified as a protective factor against behavioural sensitization to heroin addiction in a mouse model **[7]**.

AUTS2 has also been implicated as an important gene in human-specific evolution **[8]**, and research into its function suggests that it has dual roles conferred by different regions of the protein, acting within either the cytosol or nucleus of developing neurons **[5]**. As a transcription factor, AUTS2 acts as part of a novel Polycomb repressive complex (PRC 1.5), capable of genetic transactivation **[9]**, which is facilitated by an interaction between AUTS2 and the histone acetyltransferase P300 **[4]**. A conserved region within the C-terminus of AUTS2 (404–913) is critical for its nuclear function **[9]**. The cytosolic function of AUTS2 involves the stimulation of the small guanine exchange factors, DOCK1:ELMO2 and PREX1. This interaction stimulates RAC1 activity, inducing lamellopodia and aiding neuronal migration **[5]**. The cytosolic function is dependent on Proline rich region 1 (PR1; 287–470) within the N-terminus of AUTS2 **[5]**. Other predicted functional elements within AUTS2 include two conserved nuclear localising signals (NLS; NLS1–11–27 and NLS2–70–79), a WW-binding motif (PPPY; 515–519), a hexanucleotide repeat (HQHQ; 525–540), a trinucleotide repeat (Polyhistidine; 1126–1133), and two phosphorylatable serine residues (S1198 and S1233; PhosphoSitePlus IDs: 18908927 and 5207742) **[8]**.

AUTS2 is predicted to exist as part of a gene family, with Fibrosin (FBRS) and Fibrosin-like protein 1 (FBRSL1) **[10]**, referred to as the ‘AUTS2 family’. These are thought to be an ohnolog gene family, a group of duplicated genes (paralogs) generated from an ancestral progenitor through two rounds of whole genome duplication (2R-WGD), predicted to have occurred ~470 Mya (million years ago) during the evolution of jawed vertebrates (*Gnathostomes*) **[11]**. 2R-WGD gene families would have originally consisted of a group of four duplicates which, through the course of evolution, diverged into: pseudogenes (either still identifiable as inactive paralogs or unidentifiable and deemed ‘lost’), functionally distinct sequences and/or redundant sequences **[12]**. Ohnologs may have facilitated increased genomic, morphological and developmental complexity of vertebrates, for example, the expansion of the vertebrate cerebral cortex, and are associated with signalling pathways and developmental genes in vertebrates **[13]**. Retained ohnologs are also disproportionately affected by pathogenic copy number variants, have an increased susceptibility to deleterious mutations, and are frequently associated with cancer and other genetic diseases **[13–15]**.

Expression analyses in Zebrafish (*Danio rerio*) show that *Auts2*, *Fbrs* and *Fbrsl1* display distinct spatiotemporal and isoform-specific neuronal expression patterns throughout embryonic and juvenile development **[16]**. *Auts2* and *Fbrsl1* both encode C-terminal isoforms in zebrafish **[16]**; two C-terminal isoforms of Auts2 (Variants 1 and 2) are documented in mouse (*Mus musculus*), and a homolog of Variant 2 has been confirmed in humans **[2, 5]**. An N-terminal isoform of AUTS2 (AUTS2-202; ENST00000403018.2), containing an alternate exon 5, which encodes a premature stop codon, is thought to be functional and has been shown to be upregulated in a patient with a duplication within intron 4 of *AUTS2*, resulting in autism, intellectual disability and epilepsy **[17]**. FBRS also encodes a functional C-terminal isoform (FBRS-201; ENST00000287468.5) which is secreted by CD4 +ve T lymphocytes in response to ischemic myocardial infarction, where it acts as a fibrogenic cytokine, aiding in the wound healing process through promoting the differentiation of myofibroblasts **[18, 19]**. The cytokine role of FBRS has only been observed with the short isoform of FBRS (FBRS-201; ENST00000287468.5); the function of the long form of FBRS is still unknown. The function of FBRSL1 is also unknown but was previously identified as a candidate RNA binding protein **[20]**.

Although progress has been made in defining the role of AUTS2 in brain development, the function of FBRS or FBRSL1 have yet to be characterised in a neuronal context. Interaction studies show that the Polycomb group proteins PCGF3 and PCGF5 interact with both AUTS2, FBRS and FBRSL1 **[21]**, the same is also true for the Casein Kinase 2 (CK2) subunits CSNK2A2 and CSNK2B **[22]**, alluding to a level of functional, or at least mechanistic, redundancy within the family. AUTS2 and FBRS have been shown to form a complex together, but not AUTS2 and FBRSL1 **[4]**.

Genetic duplicates often retain a level of functional inheritance from their ancestral homologs **[23]**; therefore, evolutionary investigations can highlight novel animal models for the investigation of function. The C-terminal region of AUTS2 is critical to its nuclear function and shares significant homology with a *Drosophila melanogaster* protein, Tay bridge **[8, 24]**. The functional significance of the C-terminal region is supported by human studies and animal models **[2]**; it is associated with more severe forms of AUTS2 syndrome in humans, and administration of the C-terminal transcript of human *AUTS2* rescued the phenotype in a zebrafish *Auts2* MO knockdown. Due to the sequence identity displayed between AUTS2 and Tay bridge, it is possible that Tay bridge is an evolutionary relative of AUTS2, linked to the AUTS2 family through vertical transmission of the family’s ancestral progenitor, therefore, it is important to review the function of Tay bridge.

Tay bridge (Tay), a neurodevelopmental regulator, is critical to the development of the protocerebral bridge, a component of the central complex within the insect brain, analogous to the basal ganglia of humans **[25, 26]**. *Tay* mutants display an underdeveloped protocerebral bridge and complex sensorimotor deficiency featuring delayed reaction to exogenous stimuli, disordered walking pattern and slowed walking speed **[27]**. Interestingly, the phenotype displayed by *Tay* mutants is similar to that of *Auts2* MO fish, with both showing reduced responses to exogenous stimuli and impaired or disorganised movements **[6, 24]**. A functional study of Tay and AUTS2 showed that both proteins share a related, yet inverse, interaction in epidermal growth factor receptor (EGFR) signalling; wherein AUTS2 expression within laminar wing disc resulted in ectopic vein formation, while Tay overexpression produced atrophied or underdeveloped vein formation **[24]**. Tay interacts with the *Drosophila* homologs of both Mkp3 and Erk, interactions which have not previously been associated with AUTS2 **[24]**.

Here we show for the first time, a clear shared evolutionary ancestry between the AUTS2 family of proteins and Tay, highlighting Tay as a divergent homolog of the progenitor gene responsible for the generation of the AUTS2 family. This indicates *Drosophila*, or similar insect models, can be used to aid research into AUTS2 function. This is further supported by the identification of eleven regions of significant sequence identity shared by all AUTS2-related sequences, along with regions unique to each protein, which may contribute to their functional diversity. A predicted domain structure for AUTS2 was constructed, using sequence conservation data and *in silico* structural predictions, which can be used to model the effects of nucleotide variants on protein structure, and predict their potential consequences on function in a disease context. Also of interest are FBRS and FBRSL1; these proteins may be involved in neurodevelopment due to their homology with AUTS2 and distinctive neuronal expression profiles, and thus should be considered as candidates for ASD associated diseases. We also recapitulate a study into the rapid evolution of AUTS2 using population level variation data to re-examine a set of variants previously defined as *human-derived*.

## Methods and materials

### Sequence acquisition

Sequences were acquired through BLAST queries against the GenBank sequence repository **[28]**. The majority of peptide sequences used were derived from predicted gene sequences; accessed April 2017. The sequence used for human FBRSL1 (XP_005266234.1) was chosen because it lacked a likely intronic region (Hg19.chr12:133150936–133151079), included in the human RefSeq FBRSL1 sequence (NP_001136113.1). A similar sequence was constructed for Chimpanzee (*Pan troglodytes*) Fbrsl1 using genomic evidence and XP_005266234.1 as a template. The Auts2 sequence for Anole lizard (*Anolis carolinensis*) was reconstructed using an Exon 10 homolog from XP_016853289.1, which is annotated from a separate scaffold than the main GenBank gene.

### Phylogenetic analysis

Sequences were aligned using the MUSCLE algorithm **[29, 30]**. Phylogenetic analysis was performed using MEGA6 **[31]**. The evolutionary history was inferred by using the Maximum Likelihood method based on the JTT matrix-based model. Initial tree(s) for the heuristic search were obtained by applying the Neighbour-Joining method to a matrix of pairwise distances estimated using a JTT model. All positions with less than 95% site coverage were eliminated. That is, fewer than 5% alignment gaps, missing data, and ambiguous bases were allowed at any position. All presented trees were constructed with 1000 bootstraps; only bootstrap values above 70% agreement are displayed.

### Region identification

The identification of conserved exonic regions was performed using normalised conservation scores, calculated by ConSeq **[32]**. A cut-off value of 0 was used; sites ≤ 0 were treated as positive. The values were then converted into a binary plot which was smoothed with a 10-residue sliding sample script. The resultant values were plotted and peaks that scored equal 1 (average sample value) were treated as conserved regions. 18 peaks were identified within the analysis. The resultant regions were isolated from the original alignment and assessed for sequence conservation and average identity using JalView (2.11.0) and MView **[33, 34]**; regions displaying low conservation to aAUTS2p and Tay homologs were treated as false positives and removed. 11 regions were defined as true-positive conserved elements. For regions of internal conservation the process was repeated using ortholog specific alignments using: the human ortholog as the reference sequence for the AUTS2 family proteins; the sequence for Ant (*Camponotus floridanus*) for aAUTS2p; the Tay bridge sequence (*Drosophila melanogaster*) for Tay homologs.

### Sequence and structural analysis

Hydrophobicity analysis was performed in ProtScale (ExPASy) using the Miyazawa hydrophobicity scale **[35, 36]**. Disorder analysis was performed using IUPred (version 1.0) **[37]**. Disordered binding regions were assessed using ANCHOR (version 1.0) **[38]**. Linker prediction was performed using the DLP-SVM Short web service **[39]**. Disorder data was integrated with sequence conservation data, produced using the ConSeq web service, and rescaled, allowing the identification of both conserved-ordered and divergent-disordered regions. These regions along with linker analysis data were interpreted to produce a predicted domain structure. Kinase sites were predicted with Scansite 3 and preferred interacting kinase assessed with NetworKIN **[40, 41]**. Normal variation was assessed using genomic data provided by gnomAD **[42]** (accessed June 2017). Graphs were produced using OriginPro (version 8.5.1). Data used for the Neanderthal-Human-Chimpanzee analysis was accessed through UCSC using data from **Green *et al*. [43]**; the PanTro5 reference genome was used for chimpanzee and hg19 for human. Sequences were aligned using Mauve (version 1.58.0) **[44]**, nucleotides were matched using an in-house perl script and resultant graph(s) produced within the R environment (version 3.4.1).

## Results

### Interpreting the ancestry of the AUTS2 family

**Fig 1:**
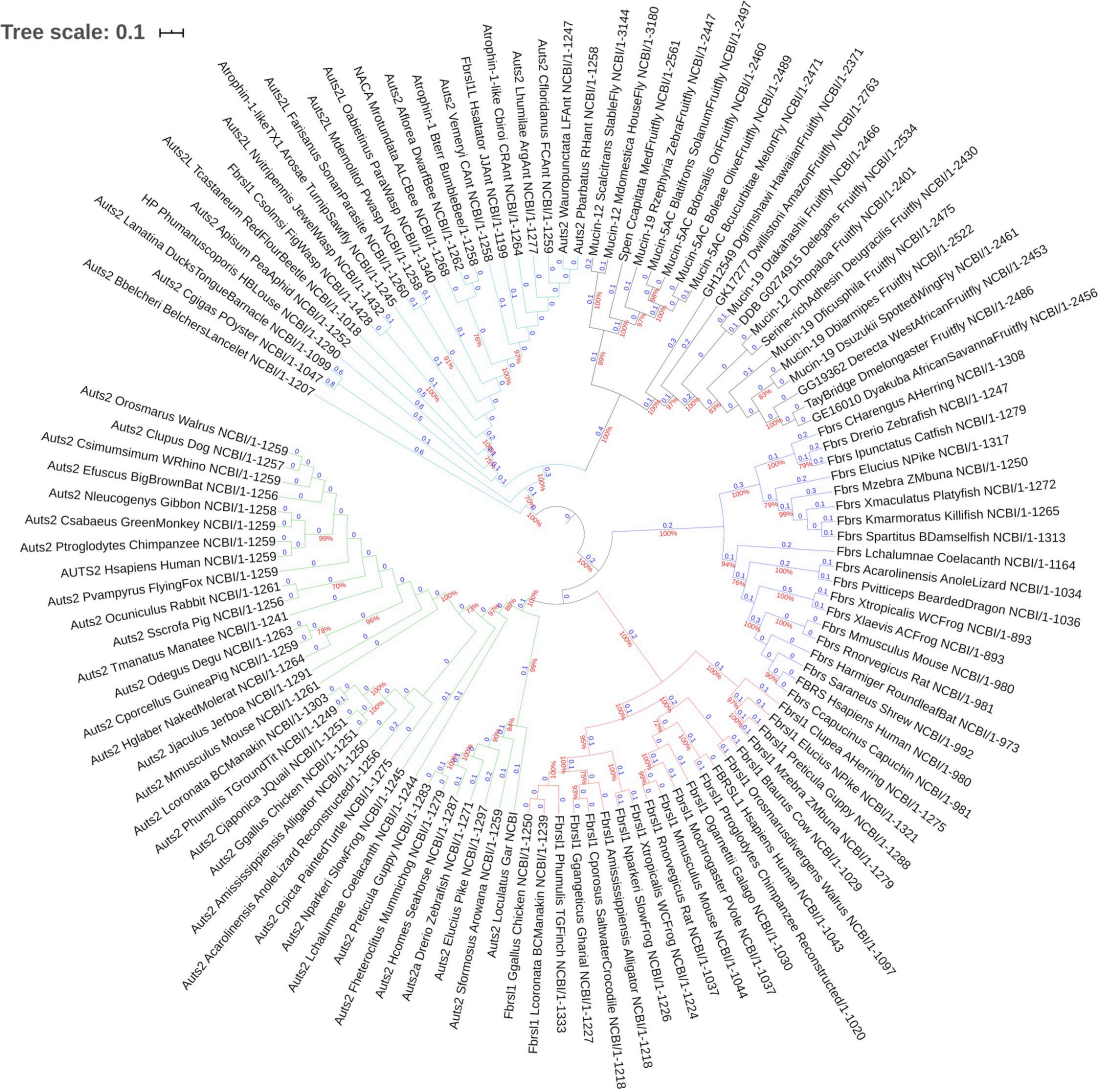
Evolutionary history of AUTS2-related proteins. Phylogenetic tree produced using MEGA6. Drawn to scale; branch lengths measured in the number of substitutions per site; 558 amino acid positions in the final dataset. 1000 bootstraps were applied; bootstrap agreement figures are displayed for clades with >70% bootstrap support. Labelling format: [Given Protein Name]_[Species: Latinised]_[Species: Common]_[Length]. Branch colour: teal: aAUTS2p; black: Tay homologs; blue: FBRS; red: FBRSL1; green: AUTS2.

A phylogenetic analysis of protein sequences displaying similarity to AUTS2 was performed; 114 peptide sequences were assessed (**S1 Table**). The results show that the AUTS2 family consists of three members, AUTS2, FBRSL1 and FBRS, within the majority of *Gnathostome* species (**Fig 1**). FBRS orthologs were not identified within any species of bird, however as FBRS orthologs are observed within reptile species, the loss of Fbrs in bird species is likely to have occurred after their divergence from reptiles. The AUTS2 family has been previously identified as an ohnolog family within the OHNOLOGS database **[10]**, supported by shared common ancestry data **[45]**. The phylogenetic analysis produced here suggests that AUTS2 is evolutionary closer to FBRSL1 than to FBRS, i.e. sharing a most recent common ancestor (**Fig 1**), which is also supported by average pairwise identity (**Table 1**; **S2–S6 Tables**). This has implications for the function and clinical relevance of FBRSL1, due to the known disease associations of AUTS2. A region within the C-terminus of AUTS2 displaying similarity to FBRS has previously been identified (Auts2 region: PF15336) **[8]**, and also displays significant similarity, inferring homology, to the *D*. *melanogaster* protein Tay bridge (Tay) **[24]**. This inferred evolutionary relatedness suggests that Tay has functional similarity to AUTS2, explaining previous research linking both AUTS2 and Tay to developmental EGFR signalling **[24]**.

**Table 1:** Averaged global pairwise identity values for AUTS2-related proteins. n = Number of sequences in each ortholog group.

AUTS2	n = 33	75%				
FBRSL1	20	42.13%	66%			
FBRS	19	32.23%	29.10%	53%		
aAUTS2p	22	23.78%	21.90%	18.99%	46%	
Tay Bridge	20	12.76%	12.18%	11.03%	18.05%	53%
		AUTS2	FBRSL1	FBRS	aAUTS2p	Tay Bridge

A 2R-WGD gene family would be represented in basal species by only one gene copy. The identity of the gene retaining the *ancestral AUTS2* function is ambiguous in humans, however *Chordates* which diverged before the 2R-WGD, such as *Amphioxus* (an order of jawless vertebrates), will possess a single gene related to the *ancestral progenitor of AUTS2* (*aAUTS2p*) **[11]**. BLAST searches identified one sequence containing the AUTS2 region (Pfam: PF15336) in the *Amphioxus* species *Branchiostoma belcheri*. A single AUTS2 region-containing protein was identified within each *Protostome* species assessed, with the lowest order species being parasitic worms (*Trichuris suis*). This suggests that the ancestral progenitor gene was present in the early ancestors of the *Nephrozoa* clade of *Bilaterians*. It should be noted that the majority of aAUTS2p sequences identified were incorrectly annotated in GenBank, e.g. Atrophin-1-like in Turnip sawfly (*Athalia rosae*) (**Fig 1**).

Phylogenetic analysis of the AUTS2 region-containing sequences, characterises aAUTS2p as a largely constrained sequence within most *Arthropoda* species, with *Hymenoptera* (Ants, Bees and Wasps) and *Mollusca* species clustering together, while divergence is apparent in sequences derived from the *Diptera* order (True flies), including *D*. *melanogaster* (Tay); wherein a separate subclade is formed to accommodate Fly aAUTS2p (referred to as Tay homologs in **Fig 1**). This divergence is illustrated by the difference in protein size between aAUTS2p in Ant (*Camponotus floridanus*; 1259 residues) and Tay (*D*. *melanogaster*; 2486 residues), which is consistent across both clades (**Fig 1**). The divergence of Tay, and its homologs, highlights it as a likely functionally discrete ortholog of aAUTS2p, which is consistent with the divergent but related functionalities of Tay and AUTS2 **[24]**.

The ohnolog status within the AUTS2 family is corroborated by the observation that all AUTS2 family sequences appear to share a common ancestral sequence with *aAUTS2p* (**Fig 1**). We show that AUTS2 displays the closest resemblance to aAUTS2p by using pairwise identity figures for Lancelet (*B*. *belcheri*) aAUTS2p (AUTS2: 30.17%; FBRSL1: 25.33%; FBRS: 23.41%), and thus may display the closest functional resemblance to aAUTS2p. To expand on the relationship between these proteins, constrained regions were identified and analysed.

### Identification of conserved regions shared by AUTS2-related proteins

**Fig 2:**
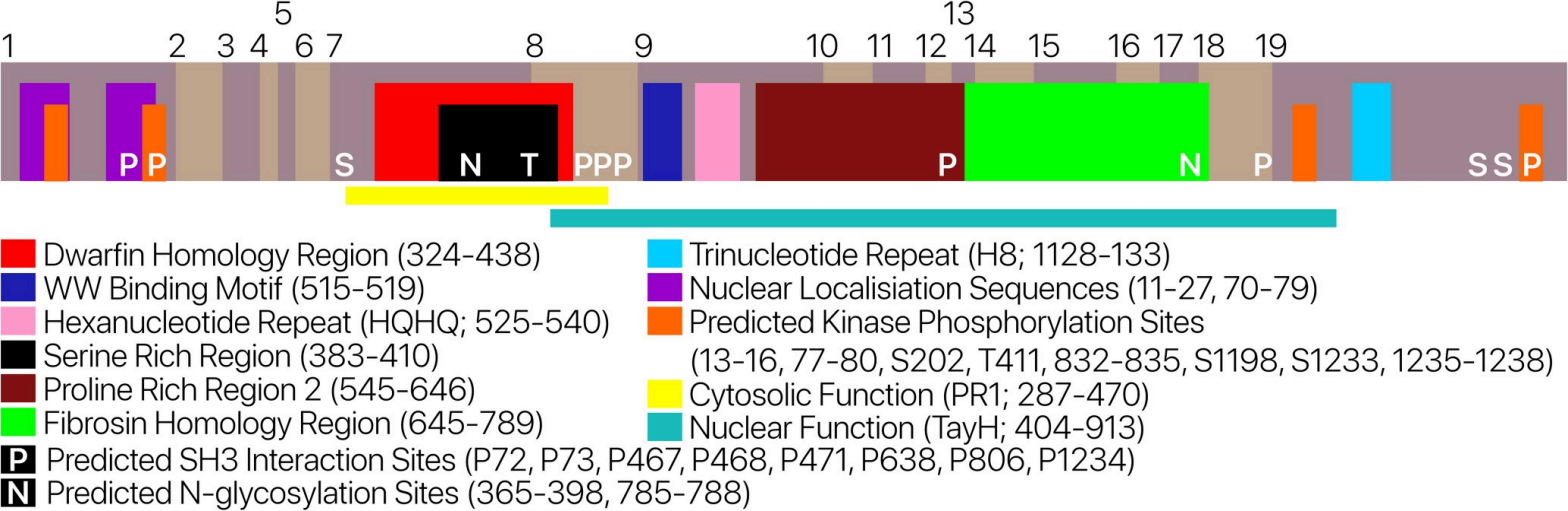
Schematic diagram of AUTS2 displaying previously annotated regions. Regions, motifs and predicted sites were attained through literature and database searches; only conserved predicted sites are displayed, non-conserved (interacting residue is not present in 100% of a 30-way AUTS2 multiple sequence alignment) sites were omitted. Conservation values for regions and motifs are displayed in **S7 Table**.

The AUTS2 protein sequence was characterised using a variety of *in silico* tools to search for motifs and predicted regions of homology. Within the Pfam database only the AUTS2 region (PF15336) was identified with significant similarity. Further regions of homology were predicted using MOTIF, however, these regions displayed either insignificant similarity or alignment to a repetitive region and were excluded from the analysis. AUTS2 nuclear localising sequences (NLS) were re-assessed using NucPred and cNLS Mapper **[46, 47]**. NucPred highlights NLS1 and NLS2 as significant, while cNLS Mapper identified all three NLS motifs (1–3). Predicted regions of importance and motifs previously highlighted in AUTS2 **[6]** were assessed for conservation across AUTS2 orthologs (**Fig 2; S7 Table**). Regions displaying high conservation include: FbrsHR, PR2, and TayHR. Based on conservation, the motifs most likely to be functional include: WW-binding Motif, hexanucleotide repeat, trinucleotide repeat, NLS1 and NLS2. We did not identify similarity to either TOP1 (human topoisomerase) or Dwarfin, so these regions were excluded from our analyses. This list does not preclude the functionality of other predicted sites but catalogues the sites of high conservation.

**Fig 3:**
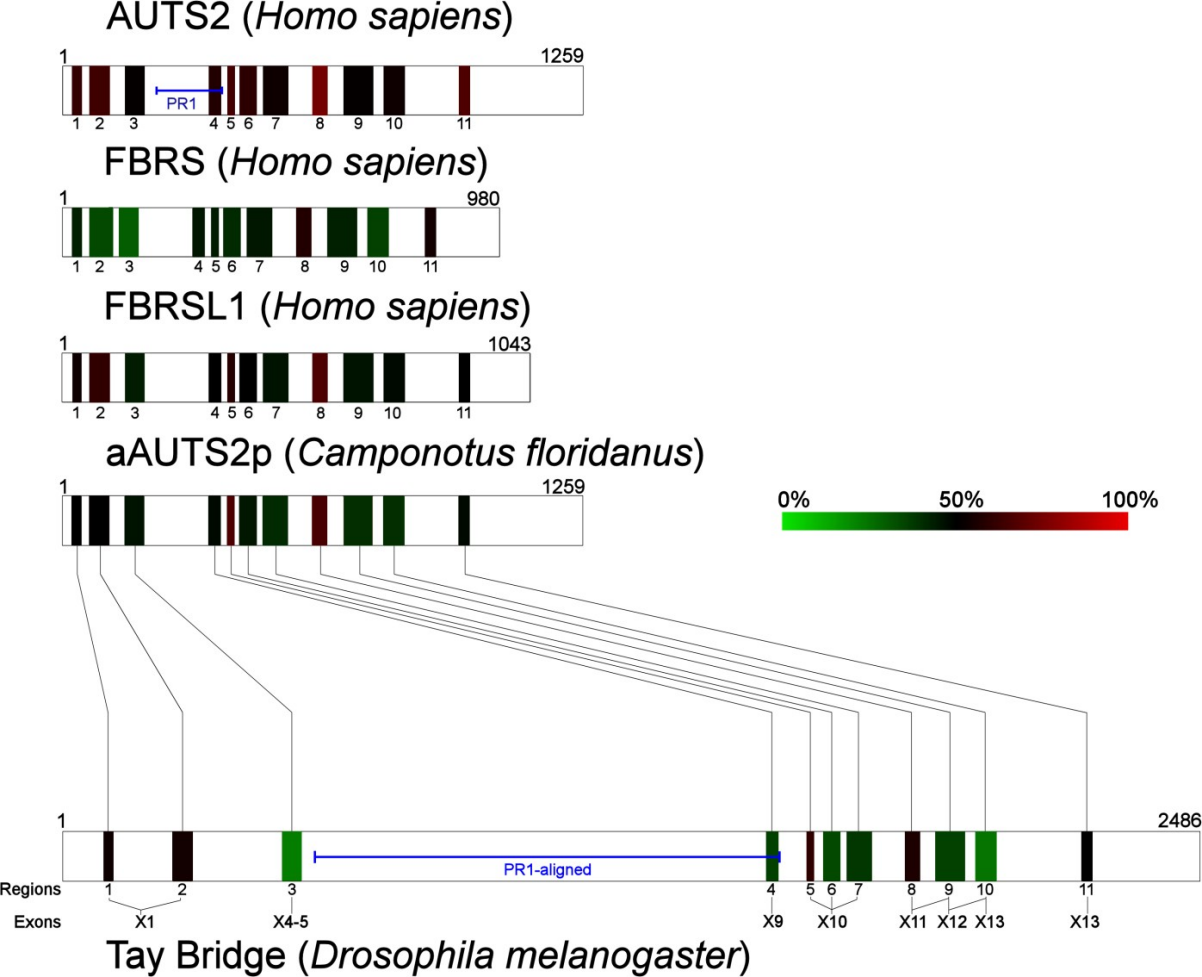
Shared regions of conservation between AUTS2-related proteins (R1-11). Heat map colour coding displays the average pairwise identity of each protein region between AUTS2-related proteins (107 sequences) as calculated by MView **[34]**. Connecting lines represent the arrangement of conserved regions in Tay bridge compared to aAUTS2p (*C*. *floridanus*). Amino acid positions are displayed above each protein. PR1: Proline rich region 1. Percentage identity values are displayed in **Table 2**.

Analysis of multiple AUTS2-related protein sequence alignments (AUTS2 family, aAUTS2p and Tay homologs) identified eleven discrete regions of high relative sequence identity (Regions 1–11; **Fig 3**), that may be of functional importance. These regions exist in the same order within all sequences assessed and include aligned regions within the N-terminus of both AUTS2 and Tay (**Table 2**; **Fig 3**; pairwise identity values in **S2–S6 Tables**). This finding elaborates on data from **Molnar *et al*. *2013***, by extending the known homology between AUTS2 and Tay to a whole protein level. The most highly conserved element aligns with exon 14 of *AUTS2* (Region 8; **S7 Table**). Interestingly, Regions 5–7 all exist within the same exon in Tay (exon 10) but are distributed across three exons in all AUTS2 family proteins (**Fig 3**). Region 6 contains the PPPY motif, a motif capable of binding to WW domains, and the HQHQ repeat region, a conserved repetitive tract with no known functionality. The PPPY motif is present in all AUTS2 orthologs and in the majority of aAUTS2p sequences (18/21; 85%), but not FBRS, FBRSL1 or Tay homologs. The PPPY motif in aAUTS2p orthologs appears preferentially in its degenerate ‘PPxY’ form, a potentially active WW-binding motif **[48]**, suggesting functionality (see **Supplementary Results in S1 File** for full details).

**Table 2:** Conserved regions within AUTS2.

	AUTS2				Pairwise Identity (%)				
	(*Homo sapiens*)				Average	FBRS	FBRSL1	Tay bridge	aAUTS2p
Region^a^	Start	End	Exon	Length (aa)	(All seq.)^b^	(*Homo sapiens*)	(*Homo sapiens*)	(*Drosophila melanogaster*)	(*Camponotus floridanus*)
Global	1	1259	1–19	1259	39.3	27	33	10	18.8
1	11	26	1	16	64.4	47.1	66.7	52.9	41.2
2	69	105	1	37	66.4	33.3	70.3	52.6	48.8
3	199	240	3–6	42	54.6	31.8	54.8	22.2	50
4	457	476	8	20	61.3	62.5	57.9	34.8	45
5	490	505	9	16	67.5	50	62.5	57.1	62.5
6	516	551	9	36	63.0	48.3	57.5	26.3	57.1
7	564	610	10–11	47	57.6	57.4	58.8	25.4	24.6
8	645	666	14	22	76.6	66.7	75	46.9	75
9	727	769	16–17	43	54.3	50	51.2	23.4	36.7
10	778	811	18	34	58.2	40	67.6	17.6	36.1
11	979	991	19	13	69.0	72.7	61.5	43.8	46.2

^a^ All residue values relate to the peptide sequence of AUTS2-201 (ENST00000342771.8).

^b^ Average identity values calculated across an alignment of AUTS2-related proteins (114 sequences).

The majority of differences between Tay and AUTS2 are localised to Proline rich region 1 (PR1) (**Fig 3**), thought to be responsible for the cytosolic function of AUTS2 **[5]**, and is divergent between AUTS2 orthologs (**S7 Table**). Sequence divergence within PR1 is largely restricted to its N-terminus (AUTS2 296–368), while the C-terminus, overlapping the Serine Rich and Dwarfin Homology regions **[8]**, is relatively conserved between species making it a more likely candidate for protein-protein interaction.

Interestingly, aAUTS2p and Tay homologs do not contain a region homologous to exons 12–13 of AUTS2, indicating that it may have emerged more recently. To identify regions likely to contribute to functional diversity between the AUTS2-related proteins, conservation was assessed within each ortholog group individually.

### Internally conserved regions display major crossover between AUTS2-related proteins

**Fig 4:**
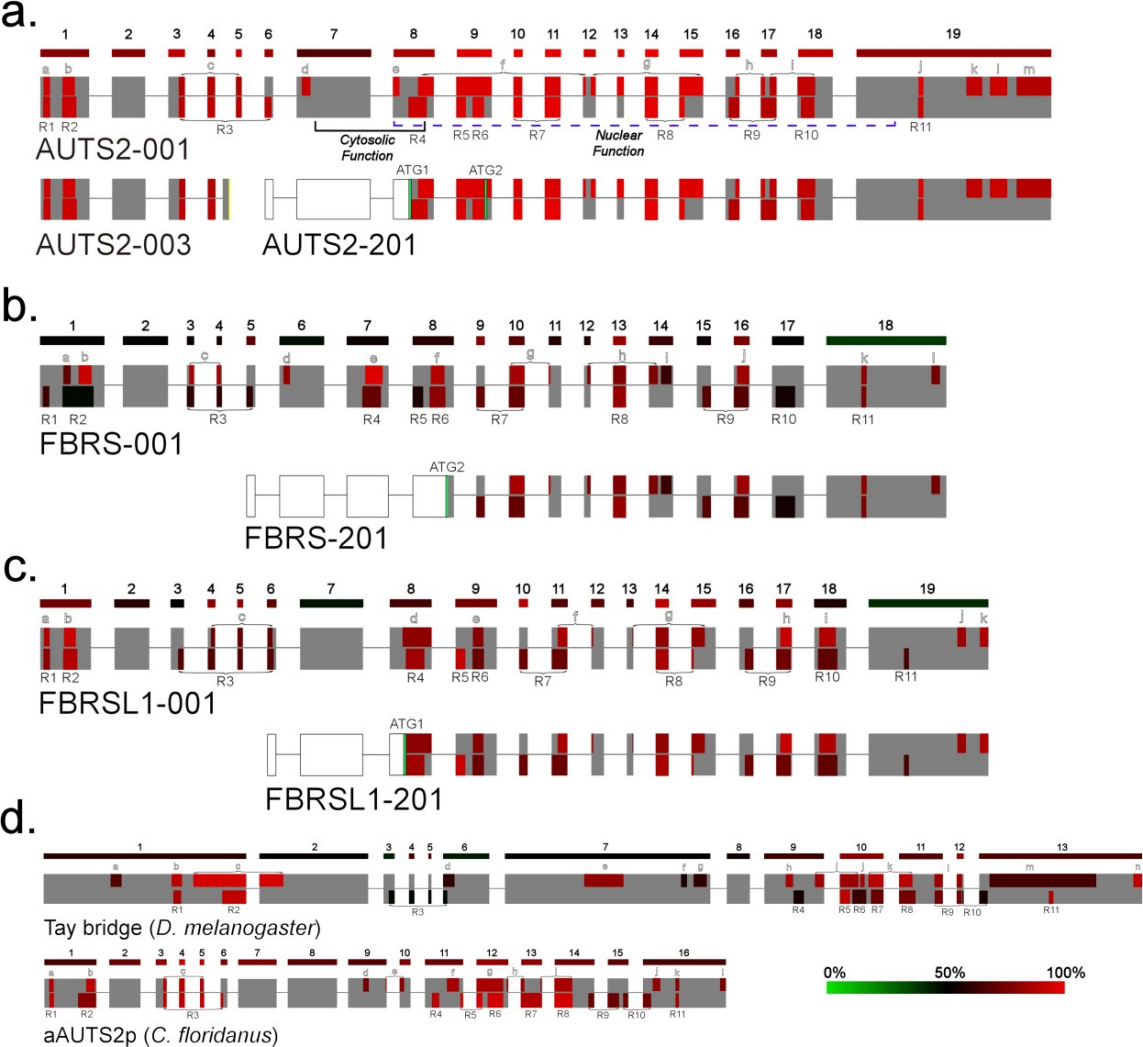
Internally conserved regions in AUTS2-related proteins. Internally conserved regions are displayed above the midpoint and those regions identified as conserved across all AUTS2-related proteins are displayed below; an exon-by-exon conservation schematic is displayed above each primary isoform. Average conservation of each region within the respective ortholog grouping is represented by a heat map. ATG1 and ATG2: start codons used by AUTS2 Variants 1 and 2 respectively. **a.** AUTS2-001: canonical isoform containing all exons; regions critical to both the *Nuclear* and *Cytosolic* are displayed with the blue dashed and black solid brackets respectively. AUTS2-003: N-terminal isoform containing exons 1–4 of the canonical transcript and an alternate exon 5 containing a premature stop codon (displayed in yellow); **b.** FBRS-001: long form of FBRS containing 18 exons, no analogous exon analogous to AUTS2 Exon 3 is present. FBRS-201: predominant C-terminal isoform of FBRS (ENST00000287468.5) analogous to AUTS2 Variant 2 and contains the majority of the Tay. **c.** FBRSL1-001: canonical FBRSL1 isoform. FBRSL1-201: C-terminal isoform containing exons 8–19; validated in zebrafish and is analogous to Auts2 Variant 1 (AUTS2-201). **d.** Schematic of Tay bridge (*D*. *melanogaster*) and aAUTS2p (*C*. *floridanus*).

Regions of high relative conservation were identified within each ortholog group individually (AUTS2, FBRS, FBRSL1, aAUTS2p, and Tay), referred to here as ‘internally conserved regions’ (**Fig 4A–4D; S8A–S8E and S9–S13 Tables** contain individual pairwise identity values). Each ortholog group was also analysed for exon by exon identity (**Fig 4A–4D**).

This analysis highlighted a large overlap of conserved regions between the AUTS2 family ohnologs, along with unique elements largely localised to PR1 and the C-terminal regions, which may contribute to functional diversity (**S8 Table**). Conservation within the AUTS2-related proteins follows a consistent pattern and can be broadly split into three domains: (i) N-terminal domain (NTD) (AUTS2; exons 1–7), (ii) Tay domain (TayD), containing the Tay homology region **[24]** (exons 8–18), and (iii) C-terminal domain (CTD) (exon 19). A region of divergence corresponding to the N-terminus of PR1 (exon 7) separates the NTD and TayD, and displays relatively low sequence conservation but retains its high proline composition. A region of high variability was also observed within the N-terminus of the CTD, referred to as the RERE repeat region, due to its high concentration of charged polar residues, which displays consistently low sequence conservation but retains its composition of alternating positive (arginine/lysine) and negatively charged residues (glutamate/aspartate).

### Characterisation of the N-terminal domains (NTD)

**Fig 5:**
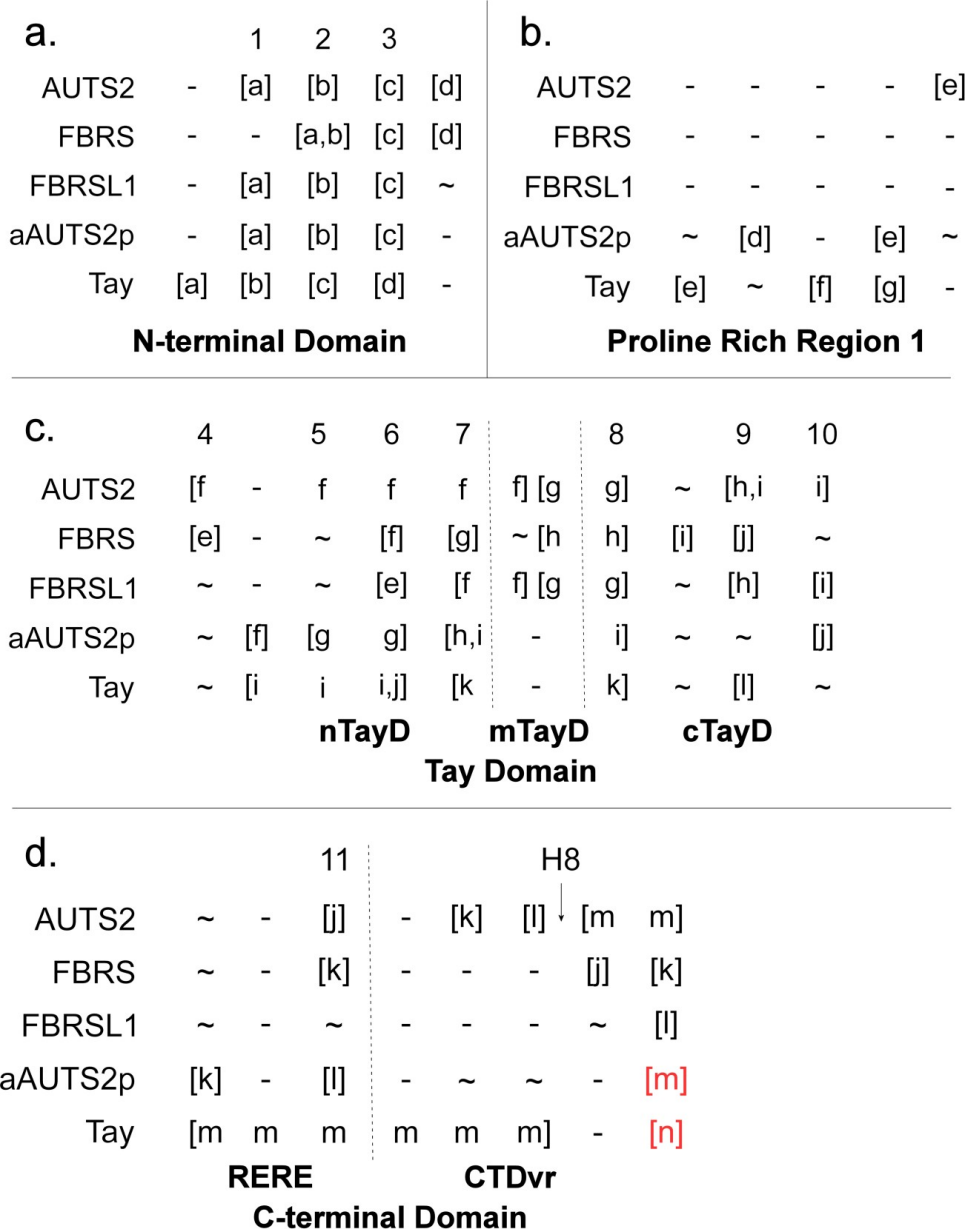
Alignment of internally conserved regions within AUTS2-related proteins. Regions aligned to R1-11 (shared conservation) are displayed above each matrix. []: internally conserved regions; divergent regions of conservation are in red; **~**: not highly conserved;—: no alignment or alignment to a region of low conservation. **H8**: octahistidine tract/trinucleotide repeat specific to AUTS2 orthologs. **RERE**: RERE repeat region; **CTDvr**: C-terminal domain variable region.

The NTD of AUTS2-related proteins display a distinct pattern of localised conservation and divergence (**Fig 5A**). Regions 1–3 overlap with regions of internal conservation, except for Region 1 of both FBRS and Tay (**Fig 5A**; **S8A–S8E Table**). The sequence between Regions 1–3 is largely divergent in all AUTS2-related proteins, excluding Tay bridge in which a large region of internal conservation lies upstream of Region 2 *(Region c*; **S8E Table**). A ~30 residue glutamate-rich insertion was identified within Region 2 of mammalian FBRS orthologs, splitting Region 2 into *Regions a* and *b* (**Fig 4**), corresponding to NLS2 and a hydrophobic tract respectively; as this disruption of Region 2 is unique to mammalian FBRS it likely represents a recent sequence insertion within the FBRS sequence.

The NTD is generally a divergent domain, although the retention of conserved elements within it, such as Region 3 and the hydrophobic portion of Region 2 suggests functionality. Of the AUTS2 family ohnologs, the NTD of FBRS is most divergent, which may be caused by C-terminal isoform predominance in FBRS; i.e. the N-terminal function is required in fewer contexts due to isoform predominance, therefore, relaxed constraints lead to increased divergence.

### Proline rich region 1 (PR1) acts as a linker between the N-terminal and Tay domains

The N-terminus of PR1 (nPR1; 296–368 AUTS2) is consistently divergent across the AUTS2-related proteins (**Fig 5B**). *In silico* prediction with DLP-SVM identifies nPR1 as a candidate linker region spanning between the NTD and TayD **[39]**, consistent with its low conservation. *Region e* of AUTS2 is relatively well conserved across AUTS2 orthologs and so probably represents the functional region of PR1. *Region e* of AUTS2 does not share conservation to Tay, FBRS or FBRSL1 orthologs, although it does display moderate conservation to the aligned region within aAUTS2p orthologs suggesting a shared cytosolic function.

In contrast, Tay homologs contain four unique regions of high internal conservation within the sequence aligned to PR1 (*Regions e-h*; **Fig 5B**; **S8E Table**); these regions display limited conservation to aAUTS2p orthologs and may represent additional functional regions within PR1 of Tay and aAUTS2p homologs, which are not present in AUTS2 family proteins.

### The Tay domain is highly conserved across the AUTS2-related proteins

The Tay domain (**TayD**) displays relatively stable conservation across all AUTS2-related proteins (**Fig 5C**), however the N-terminus of Region 6, containing the WW-binding motif in AUTS2, is unique in each ohnolog, potentially representing a region of functional divergence within the human homologs, though as previously noted some aAUTS2p homologs retain the WW-binding motif.

The TayD can be divided into three discrete subdomains: nTayD (AUTS2 exons 9–11), mTayD (AUTS2 exons 12–13) and cTayD (AUTS2 exons 14–18). There is no sequence similarity for the mTayD in aAUTS2p and Tay orthologs. Exon 11 is spliced out of rodent *Fbrsl1*, but not human FBSRSL1, due to it containing a premature stop codon suggesting that exon 11, and potentially the whole nTayD, may not have functional importance.

### The C-terminal domain (CTD) contributes to functional divergence within the AUTS2 family

The CTD of AUTS2-related proteins are the primary regions of divergence between AUTS2-related proteins (**Fig 5D**). This domain consists of two sub-regions, we have named ‘RERE repeat region’ (RERE; AUTS2 812–991), and ‘CTD variable region’ (CTDvr; 992–1259). RERE (N-terminus of exon 19 to Region 11) is highly divergent within the AUTS2 family and aAUTS2p orthologs but is conserved within *Region m* within Tay orthologs. CTDvr displays high levels of internal conservation within AUTS2 homologs and is truncated in both mammalian FBRSL1 and FBRS. This region may act as a variable domain contributing to functional divergence between each AUTS2 family member. The marked truncation of the CTD in the mammalian orthologs of both FBRS and FBRSL1 is illustrated by comparing the pairwise identity of AUTS2 CTD and Human FBRSL1 (27.8%) to that of AUTS2 and Chicken (*Gallus gallus*) Fbrsl1 (42.79%). The CTD of Tay is moderately conserved across the ortholog group, with a large region of conservation (*Region m*) included within the RERE repeat region suggesting a possible ancestral function for this region, potentially lost in AUTS2 family ohnologs. *Region m* of Tay displays similarity to the DISC-1 interacting region of TRAF3 Interacting Protein 1 (TRAF3IP1), a microtubule interacting protein involved in TNF signalling; predicted using MOTIF.

The CTD of AUTS2 contains four regions of high internal conservation (*Regions j-m*); *Region j* aligns to Region 11 and *Regions k-m* represent highly conserved elements within the CTDvr (**Fig 5D**). The sequence aligned to *Regions k-l* of AUTS2 is conserved in both non-mammalian FBRS and FBRSL1 orthologs but has degraded in the representative mammalian sequences. The extreme C-terminus of the CTD (*Region m* in AUTS2, *Region l* in FBRS, and *Region k* in FBRSL1) is well conserved across the AUTS2 family and contains both phosphorylatable serine residues of AUTS2 (S1198 and S1233). This region may be involved in dynamically regulating protein function through phosphorylation. The predicted interacting kinases for AUTS2 S1198 are ERK1/2, and once phosphorylated this residue is predicted to interact with PIN1, and the predicted interacting kinases for S1233 are ERK1 and PKCα/β (predicted using NetworKIN **[41]**).

The polyhistidine repeat within the CTD of AUTS2, predicted to promote protein localisation within nuclear speckles **[8]**, is not present within AUTS2 orthologs derived from fish species, although a shorter tract containing six histidine residues is present within Arowana (*Scleropages formosus*; a species of mouthbrooder). This may highlight a degradation of the tract in the majority of fish species, and a subsequent re-evolution of the tract within the Arowana species mentioned. Natural variation within the polyhistidine tract of AUTS2 is notable in humans, with homozygous histidine duplications existing within the ExAC/gnomAD variant population database **[42]**. Further *in silico* structural analysis was performed to annotate potential structural features of AUTS2-related proteins.

### AUTS2 family proteins conform to a ‘three domain’ structure

**Fig 6:**
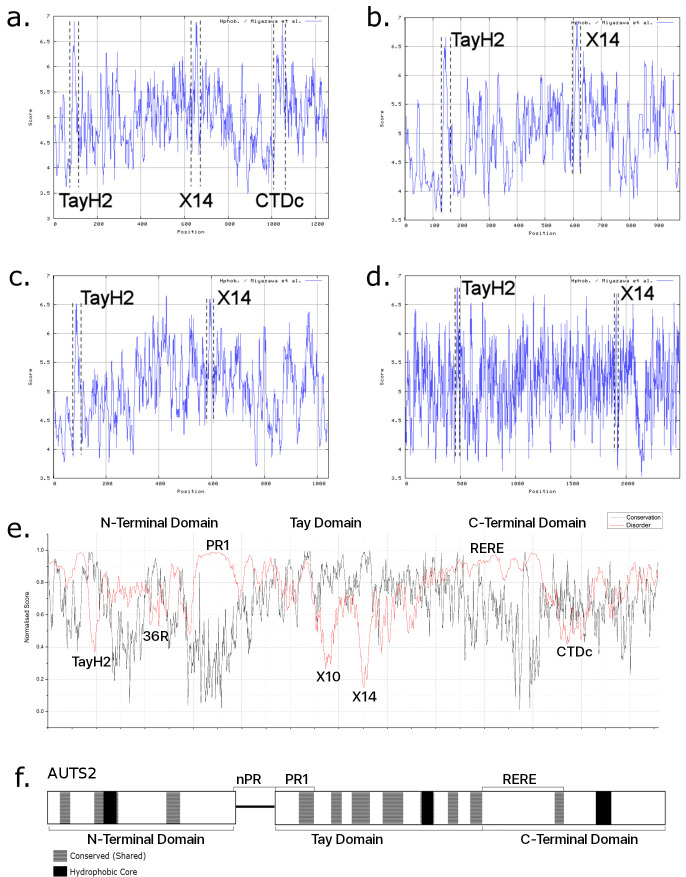
**Putative domain structure for AUTS2. a-d:** hydrophobicity plots. **a.** AUTS2; 3 hydrophobic cores: TayH2 is present in all AUTS2-related proteins; X14 (Exon 14) is the most conserved region of AUTS2 and displays a peak in hydrophobicity in all AUTS2-related proteins; CTDc (C-terminal Domain Core) is not conserved in FBRS, FBRSL1 and Tay. **b.** FBRS. **c.** FBRSL1; a peak is also visible at ~400–410 which is located between Regions 4 and 5. **d.** Tay bridge; the distance between both cores in comparison to the AUTS2 family proteins should be noted. **e.** Line graph displaying conservation across AUTS2 along with disorder potential. Regions of high conservation and low disorder potential: TayH2 (Region 2), 36R (Region 3), X10 (Exon 10; Region 6), X14 (Region 8) and CTDc. Regions of low conservation and high disorder potential: PR1 and RERE repeat region. **f.** Revised three domain structure for AUTS2.

*In silico* protein structure analysis using ProtScale-ExPASy and the Miyazawa hydrophobicity scale **[36]** identified three potential hydrophobic cores within AUTS2; two are shared between all AUTS2-related proteins, Tay Homology Region 2 (TayH2; 84–103; hydrophobic tract within Region 2), and exon 14 (645–668; Region 8)) (**Fig 6A–6D**). These putative hydrophobic cores may aid the folding and stability of the domain structure within all AUTS2-related proteins. A third predicted core within AUTS2 (1019–1112; C-terminal Domain core; CTDc; Region *k*) is not well conserved in either FBRS or FBRSL1, although non-mammalian Fbrsl1 and aAUTS2p homologs do display limited conservation, as stated previously.

Analysis of predicted disorder, calculated by IUPred **[37]**, within the AUTS2 peptide sequence indicates that AUTS2 contains intrinsically disordered regions within its N- and C-termini, with a largely ordered central domain. Regions of high conservation and low disorder potential were identified (**Fig 6E**), annotated as: TayH2 ((Tay Homology Region 2; *Region 2*), 36R (*Region 3*), X10 (exon 10; *Region 6*), X14 (Exon 14; *Region 8*) and CTDc (*Region k*); along with regions of low conservation and high disorder potential: nPR1 and RERE repeat region. Disordered binding regions are predicted to occur across 64.4% of the AUTS2 protein (see **S14 Table**).

Using the structural data described here, it was possible to predict a domain structure for AUTS2 including three main domains and a disordered linker region (**Fig 6F**), consistent with the results produced by conservation analysis: **(i)** the N-terminal domain (1–295), a largely disordered domain containing TayH2 (Region 2) as a predicted structural core; **(ii)** a highly disordered region comprising nPR1 (296–368); **(iii)** the Tay Domain (396–824), the most ordered region of AUTS2, containing exons 10 and 14 (*Regions 6* and *8*) as a structured core element; and **(iv)** the C-terminal domain (825–1259), containing the intrinsically disordered RERE repeat region followed by a predicted ordered region with CTDc, acting as a potential structural core.

### Sequence variation within the AUTS2 family in human populations

Mutations in *AUTS2* cause a human developmental disorder, AUTS2 syndrome, we therefore investigated variant load in large control human populations for each *AUTS2* family gene. Missense variant distribution and density was assessed using data acquired from the gnomAD variant database (v2.1.1), comprising whole-genome/exome sequences from 15,496 unrelated individuals **[42]**. Peaks in normal missense variation, representing higher variant density, are largely comparable between the different AUTS2 family proteins, consistently occurring within both the nPR1 and RERE repeat regions, which are largely divergent between species (**S1A–S1C Fig in S1 File**). Interestingly, a peak in variation also occurs within the highly conserved *Region m* (*Rm*) of AUTS2 and within the mTayD, indicating that these regions are less constrained, or more tolerant to variation (**S1A Fig in S1 File**). Of note, other regions with a high density of variants include the nTayD region in FBRSL1, consistent with the loss of Exon 11 in rodent Fbrsl1 (**S1B Fig in S1 File**) and the NTD in FBRS, which is consistent with the previously mentioned, inferred lack of N-terminal constraint (**S1C Fig in S1 File**).

In general, non-conserved residues within each protein show more variation **[49]** and, based on our analyses, in AUTS2 it is 2.6 times more likely that a missense variant will affect a non-conserved residue compared with a conserved residue (odds ratio; OR = 2.62). For FBRSL1 missense variants are 1.5 times more likely to occur in non-conserved sites (OR = 1.47) and 1.2 for FBRS (OR = 1.22). This ratio is far lower than for AUTS2, which may imply that they are more tolerant to nucleotide variation and consequently less likely to have important roles in disease. Gene variation intolerance metrics such as GeVIR **[50]** confirm that AUTS2 is more intolerant to missense variation that either FBRS or FBRSL1 (15.07, 72.96 and 89.31% respectively).

### *AUTS2* and human evolution

*AUTS2* was implicated as a rapidly evolving gene by **Green *et al*. [43]**, who compared biallelic positions in six human genomes with the aligned position in three Neanderthals (*Homo neanderthalensis*) and the chimpanzee reference genome. The resulting analysis identified sites in which the Neanderthal nucleotide matched the chimpanzee but not the majority of the human genomes (*human derived* alleles), highlighting them as possible points of human divergence from Neanderthal. Sampling across the whole genome identified the N-terminus of *AUTS2* as containing the most *human derived* alleles, implicating *AUTS2* as a rapidly evolving gene and a potential accelerator of human-specific evolution.

With the advent of large population genome databases (e.g. gnomAD **[42]**, containing 15,708 whole human genomes) it was possible to reassess the variant allele frequencies for each of the 66 significant sites identified by **Green *et al*.** within *AUTS2* (**S15 Table**). To classify the sites, the reference nucleotide (human genome build hg19) at each position was compared to that of chimpanzee (PanTro5), where the sites matched this position was classified as ancestral or conversely as derived. Assessing the allele frequency at each site using gnomAD data showed that ancestral sites (12/66) displayed a tendency towards high frequency SNP alleles (allele frequency (af) > 0.5) with a median frequency of 0.62 (alternate *human derived* allele present in >62% of the gnomAD population; **S2 Fig in S1 File**). The site at chr7:69188495-G-T (rs73170834; af = 0.0989) represents the most *fixed* chimpanzee allele, with the *human derived* allele present in less than 10% of the gnomAD population, indicating that it cannot be confidently linked to human-specific evolution.

Each derived site contained a SNP matching the chimpanzee allele; the allele frequencies of these sites varied widely but displayed a median of 0.28 (chimpanzee allele present in ~28% of the gnomAD population) (**Fig 1**). The only derived site with a chimpanzee allele present in less than 5% of the population was chr7:70077905-A-G (rs4717538; af = 0.0147), where G matches the aligned chimpanzee allele; this site exists within a TCF7L2 binding site identified by ENCODE. rs4717538 exists within intron 5 and occupies a nucleotide base that is not conserved well between species; the importance of this finding is difficult to interpret without any further data.

## Discussion

This work characterises the AUTS2 family as a tripartite ohnolog family. A divergent ortholog of aAUTS2p is found in modern fly species, e.g. *D*. *melanogaster*, with the name Tay bridge. This divergence is consistent across the *Diptera* (True flies) order, differentiating aAUTS2p in *Diptera* from that of *Hymenoptera* (sawflies, wasps, ants and bees) and other *Arthropoda* species, due to a large expansion in sequence length. It should however be noted that within the *Diptera* order, flies of the *Brachycera* suborder (including *Drosophila*) display longer sequence lengths (~2500 residues) than those of the *Nematocera* suborder (including mosquitos and crane flies; ~1700 residues), which is still significantly longer than those of *Hymenoptera* (~1250 residues). This places the initiation of sequence expansion in aAUTS2p and subsequent evolution of Tay as early as the Permian geological period, 250 Mya, during the speciation of the *Diptera* order **[50]**. While Tay is a divergent member of the extended AUTS2 family, it retains regions within both its N- and C-termini (eleven identified in this study) displaying high sequence identity to aAUTS2p and AUTS2 family orthologs, inferring homology through vertical transmission.

Tay is a large protein (2486 residues) in comparison to AUTS2 (1259 residues), and the majority of the sequence expansion aligns to the highly divergent PR1 region of AUTS2, predicted to contain an inter-domain linker and potential disordered binding regions. Whether the expanded PR1 in Tay acts as a linker or has gained additional functionality is unknown, however constrained regions within the sequence expansions of Tay (N- and C-terminal domains *and PR1 Regions e*, *f*, *g*) may contribute to functional divergence between AUTS2 and Tay **[24]**.

Tay may also be involved in neurodevelopment but, due to the apparent sequence divergence and functional evidence provided by **Molnar *et al*.**, showing that AUTS2 and Tay display a related but converse interaction within developmental EGFR signalling, it is apparent that their functionalities are not equivalent. Tay could have acquired a different function to AUTS2 and non-fly aAUTS2p homologs through sequence expansions. As non-fly aAUTS2p orthologs share a similar sequence length to AUTS2, along with a potentially functional WW-binding motif, they may share a similar function, a hypothesis supported by our observation that AUTS2 is the closest family member to aAUTS2p in terms of sequence identity. Further research identifying the phenotype of an *aAUTS2p* knockout would be useful in defining the predicted functional divergence of Tay and the ancestral role of aAUTS2p. Although the function of aAUTS2p is unknown, it is common for duplicated genes to retain a similar function to their ancestral sequence. The existence of conserved regions within the Tay domain of AUTS2-related proteins, the region predicted to be responsible for the nuclear function of AUTS2, suggests that aAUTS2p, Tay, FBRS and FBRSL1 may also act as transcription factors through an interaction with Polycomb group proteins.

Our results show that sequence divergence between AUTS2-related proteins is complex. The conserved regions, not shared by other homologous sequences, may contribute to functional divergence. Divergence within the AUTS2 family is most readily displayed in the PR1 region, while unique regions of conservation frequently occur within the divergent CTDs. The CTDs of mammalian FBRS and FBRSL1 orthologs are truncated and display high inter-protein divergence; it is likely that this divergence contributes to functional partitioning within the AUTS2 family. PR1 is the least conserved region of AUTS2, a consistent feature across FBRS, FBRSL1 and aAUTS2p orthologs. This region is also predicted to be intrinsically disordered, indicating that sequence conservation is not necessary for it to perform its function. The WW-binding motif in AUTS2 and aAUTS2p may also be a contributing factor to functional divergence, due to its consistent retention in some homologs but not others. Also of note is the divergence of the NTD in FBRS orthologs; while this divergence is relatively small, there may still be an effect on protein function due to the splitting of Region 2 into *Regions a* and *b*, and the high missense variant load observed within the NTD of FBRS.

Functional divergence within the AUTS2 family may also manifest in dynamic factors such as tissue-specific expression and sub-cellular localisation. Expression analysis of AUTS2 family members within Zebrafish (*D*. *rerio*) **[16]**, showed that all three proteins display discrete neuronal expression patterns, including isoform-specific expression, suggesting that the complex spatiotemporal expression of AUTS2 family proteins in human tissues reflects their diverged functions. FBRS has a cytokine function **[18, 19]**, however, whether this is related to the function of AUTS2 is unknown. What is known is that the cytokine function is associated with a C-terminal isoform of FRBS **[19]**, containing the Tay and the C-terminal domains, therefore, this function may be linked to that of the Tay domain, i.e. PRC binding.

The AUTS2-related proteins are predicted to contain regions of intrinsic disorder. Intrinsic disorder is associated with the retention of paralogs generated through whole genome duplication events but not those of small-scale duplications **[51]**. This information strengthens the apparent ohnolog relationship between the AUTS2 family members.

It is likely that the ‘three-domain’ architecture of AUTS2 described here is consistent for all AUTS2 family proteins. The ordered Tay domain is likely to be the stable core of the structure with the two variable (N- and C-terminal) domains producing functional diversity between the family members. This requires laboratory-based validation, and future structural analysis of AUTS2 should consider the predicted disorder within the N-terminal domain, PR1 linker region and CTD, as it is likely that these regions may require complex binding to produce a stable structure.

AUTS2 is also predicted to have a cytosolic function requiring the presence of the PR1 domain **[5]**. The function of the N-terminal end of PR1 is predicted here to be a linker region, which may be necessary to maintain optimum domain spacing and orientation for protein binding. If PR1 acts as a linker, the region responsible for the cytosolic function of AUTS2 is unclear, as function is often associated with sequence constraint, although intrinsic disordered binding regions can be ‘hidden’ within non-conserved sequence **[52]**. The reality may be that high disorder and lack of conservation within the region indicate the presence of an intrinsically disordered binding region, as predicted by ANCHOR **[38]**. Based on conservation and population variant density, the most likely candidate regions for conventional protein-protein interaction within PR1, exist within its moderately conserved C-terminal end (*Region e* and Region 4). However, until more precise deletion constructs are produced for functional investigations, specific regions within PR1 affecting the cytosolic function of AUTS2 cannot be accurately defined **[5]**.

The reassessment of *AUTS2* in the context of human evolution disagrees with the original findings from **Green *et al*. [43]**, in that sites identified as rapidly evolving show little evidence of purifying selection as high rates of heterozygosity exist with ancestral alleles present in the chimpanzee genome. The high SNP frequency at ancestral sites (af > 0.5; 10/12 sites) suggests that the current human reference genome does not provide a good benchmark to assess base mismatch between human and chimpanzee. The ancestral sites identified here contain *human derived* alleles which are present in over 50% of the gnomAD population, thus represent minor alleles when factoring in normal variation assessed at a large population level. In contrast, there is a lower level of ancestral alleles at some derived sites, although with a median allele frequency of ~0.3 it is difficult to assess them as being under purifying selection. The only derived site selected by **Green *et al*.**, containing a SNP that could be classified as rare (af < 0.05), would be rs4717538, present in just under 1.5% of the gnomAD population. Variant rs4717538 has not previously been associated with any disease or trait within a GWAS study, however due to its rarity it may warrant further investigation. It should also be noted that for every site selected, the matched chimpanzee allele is still present within the human population (af > 0.01), indicating that purification at these sites is not complete, so these alleles are unlikely to have contributed to human-specific evolution.

While *AUTS2* may be an important gene for neurodevelopment, it is difficult to assess the evidence provided by **Green *et al*.** as statistically powerful enough to identify any single gene as influencing human-specific evolution, a criticism conceded by the authors. The study also illustrates the unrepresentative nature of using low sample numbers (human n = 6 and Neanderthals n = 3) to assess evolutionary base mismatch, and the necessity of incorporating natural variation. The link between *AUTS2* and human-specific evolution may still exist but would require reanalysis at a genome-wide level with large human variant datasets such as those produced by gnomAD. With the recent publication of high-coverage Neanderthal genomes, the amount of data available for similar studies has increased. which could make reanalysis a possibility.

## Conclusions

Collectively our results provide a detailed evolutionary understanding of and a basis for future research into the AUTS2 family of proteins. The identification of discrete regions of sequence similarity throughout the homologs strengthens the likelihood that these proteins may have overlapping biological roles. This is supported by previous interaction studies, which show redundant binding activity between AUTS2, FBRS, and FBRSL1 with Polycomb and CK2 subunits **[4, 22]**. As ohnologs are frequently identified as disease-associated genes **[14]**, both FBRS and FBRSL1 should be investigated as potentially important proteins for future research, although they may not be as biologically important as AUTS2, due to their lower levels of internal conservation and the higher tolerance for missense variants occurring within evolutionarily conserved residues. In addition, FBRS is not present within any species of bird and, therefore, may perform either a non-essential or a detrimental function within avian biology. Due to the similarity between AUTS2 and FBRSL1, further research is necessary to assess the biological role of FBRSL1 and its possible association with human disease. In fact, renaming this gene to AUTS2L1 (AUTS2-like Protein 1) would be recommended as it shows less similarity FBRS than to AUTS2. Our results provide a framework for more targeted investigations to validate the regions of AUTS2 predicted to be functionally important. Further research into aAUTS2p may also aid our understanding of the role of the AUTS2 family and how they contributed to the complexities of modern eukaryotic species development.

## Supporting information

S1 Table (XLSX)

S2 Table AUTS2 shared regions of conservation. Pairwise identity values calculated by MView.
(XLSX)

S3 Table FBRS shared regions of conservation. Pairwise identity values calculated by MView.
(XLSX)

S4 Table FBRSL1 shared regions of conservation. Pairwise identity values calculated by MView.
(XLSX)

S5 Table aAUTS2p shared regions of conservation. Pairwise identity values calculated by MView.
(XLSX)

S6 Table Tay shared regions of conservation. Pairwise identity values calculated by MView.
(XLSX)

S7 Table Conservation of previously identified regions. Pairwise identity values of AUTS2 regions collated by Oksenberg *et al*. 2013 (Oksenberg and Ahituv, 2013).
(DOCX)

S8 Table Regions of internal conservation. A. AUTS2 B. FBRS C. FBRSL1 D. aAUTS2p (*Camponotus floridanus*) E. Tay brige (*Drosophila melanogaster*).
(XLSX)

S9 Table AUTS2 regions of internal conservation. Pairwise identity values calculated by MView.
(XLSX)

S10 Table FBRS regions of internal conservation. Pairwise identity values calculated by MView.
(XLSX)

S11 Table FBRSL1 regions of internal conservation. Pairwise identity values calculated by MView.
(XLSX)

S12 Table aAUTS2p (*Camponotus floridanus*) regions of internal conservation. Pairwise identity values calculated by MView.
(XLSX)

S13 Table Tay bridge (*Drosophila melanogaster*) regions of internal conservation. Pairwise identity values calculated by MView.
(XLSX)

S14 Table Predicted disordered binding regions within AUTS2, calculated by ANCHOR. (XLSX)

S15 Table (XLSX)

S1 File (DOCX)
