# Supplementary material for: Ancestry of the AUTS2 family–A novel group of polycomb-complex proteins involved in human neurological disease
Source: PLoS One. 2020 Dec 11;15(12):e0232101. doi: 10.1371/journal.pone.0232101 (PMC7732068; doi:10.1371/journal.pone.0232101)
Supplement: S7 Table — Pairwise identity values of AUTS2 regions collated by Oksenberg et al. 2013 (Oksenberg and Ahituv, 2013). (DOCX) [file pone.0232101.s007.docx]

**S7 Table.** **Conservation of previously identified regions.** Pairwise identity values of AUTS2 regions collated by **Oksenberg *et al*. 2013 (**[**Oksenberg and Ahituv, 2013**](#_ENREF_1)**)**.

|  |  | % Pairwise Identity | | |
| --- | --- | --- | --- | --- |
|  |  | Chimpanzee | Mouse | Zebrafish |
|  | Location | *Pan troglodytes* | *Mus musculus* | *Danio rerio* |
| Full Sequence | 1-1259 | 99.8 | 92.6 | 59.1 |
| Dwarfin Homology | 326-435 | 100 | 88.4 | 37.4 |
| Fibrosin Homology | 645-789 | 100 | 98.7 | 79 |
| Human Topoisomerase Homology | 880-920 | 100 | 82.9 | 47.8 |
| Tay Homology | 580-665 | 100 | 100 | 95.3 |
| Proline Rich 1 | 288-471 | 99.5 | 86.5 | 36.9 |
| Proline Rich 2 | 545-646 | 100 | 100 | 90.2 |
| Serine Rich | 383-410 | 100 | 88.9 | 53.6 |
| WW-binding Motif | 515-519 | 100 | 100 | 100 |
| Hexanucleotide (HQHQ) Repeat | 524-540 | 100 | 100 | 81.8 |
| Trinucleotide (H8) Repeat | 1126-1133 | 100 | 93.8 | 31.2 |
| NLS1 | 11-27 | 100 | 100 | 70.6 |
| NLS2 | 70-79 | 100 | 100 | 90.9 |
| NLS3 | 120-141 | 100 | 100 | 50 |

Oksenberg, Nir and Nadav Ahituv 2013. The role of AUTS2 in neurodevelopment and human evolution. Trends in genetics : TIG 29: 10.1016/j.tig.2013.1008.1001. doi: 10.1016/j.tig.2013.08.001
