# Supplementary material for: Ancestry of the AUTS2 family–A novel group of polycomb-complex proteins involved in human neurological disease
Source: PLoS One. 2020 Dec 11;15(12):e0232101. doi: 10.1371/journal.pone.0232101 (PMC7732068; doi:10.1371/journal.pone.0232101)
Supplement: S1 File — (DOCX) [file pone.0232101.s016.docx]

**Ancestry of the AUTS2 Family – A Novel Group of Polycomb-complex Proteins Involved in Human Neurological Disease**

Robert A Sellers^1^, David L Robertson^2^, and May Tassabehji^1*^

**
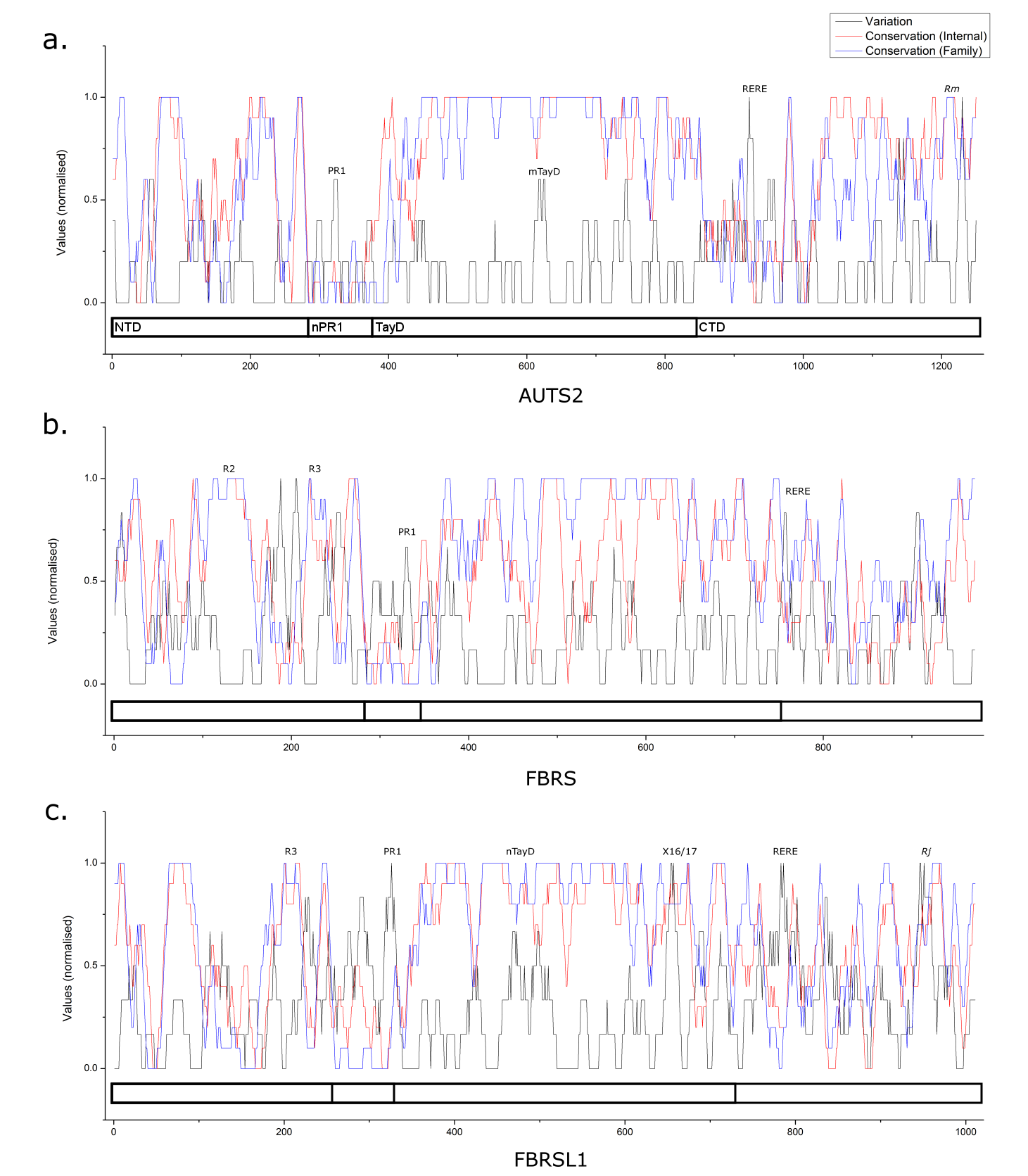
**

**S1 Fig. Graphical representation of normal variation and conservation across the AUTS2 family proteins.** Graphing data produced with a sliding sample method using binary plots to represent conserved/non-conserved and variable/non-variable positions; data was subsequently scaled and plotted using OriginPro 8.5.1. Conservation (Internal): human sequence in comparison to an alignment of orthologues; Conservation (Family): human sequence in comparison to an alignment of sequences including all AUTS2 family proteins. Population variation data was obtained from gnomAD **(**[**Lek, Karczewski et al. 2016**](#_ENREF_28)**)**; accessed June 2017. **a.** AUTS2. **b.** FBRS. **c.** FBRSL1. Abbreviations: PR1 – proline rich region 1; RERE – RERE repeat region; mTayD – medial Tay domain; nTayD – N-terminal Tay domain; X16/17 – Exon 16/17 boundary region; *Rx* – *Region ‘x’* (internal); Rx – Region ‘x’ (shared).

**
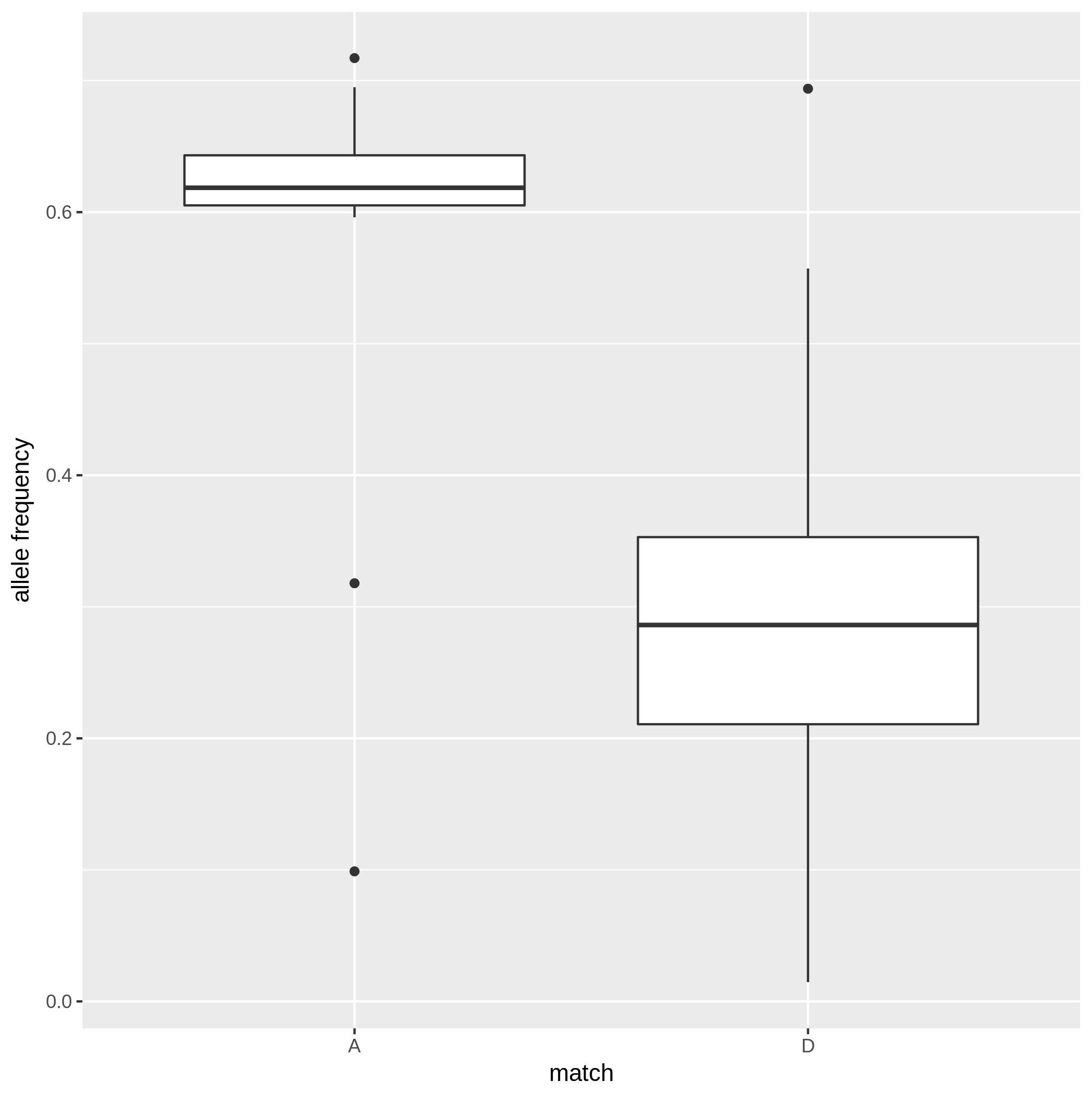
**

**S2 Fig.** Box plot displaying the allele frequency of SNPs at sites marked as either ancestral (A) or derived (D). Plot produced using ggplot2 within the R environment.

**Supplementary Results**

**Commentary on the Predicted Domains and Constrained Regions**

**N-terminal Domain and PR1 Linker Region**

The N-terminal Domain (NTD) is likely to represent a largely disordered domain encompassing Exons 1-6 of AUTS2 along with a highly conserved region (between AUTS2 family homologues) within the N-terminal of Exon 7 (266-283) prior to PR1. This domain includes the conserved NLS regions, TayH2 and the 36R. It is likely that TayH2 acts as a hydrophobic core region for the predicted NTD through due to the predicted disorder of the region it may be intrinsically disordered and only able to form a stable structure upon substrate binding.

Variation within the NTD occurs largely outside of the conserved regions though exceptions do exist. *Teleost* Auts2 homologues do not contain a homologue of Exon 4. FBRS homologues contain a genetic insert between the NLS2 and TayH2 regions. Tay contains a largely expanded NTD with large inserts occurring; prior to NLS1, between NLS1 and NLS2 and prior to 36R. Downstream of the NTD is a predicted linker region likely to link the NTD and TayD; this region in Tay is largely expanded and contains regions of both order and disorder; it may be that this region represents the major region of functional divergence between AUTS2 and Tay.

**NLS1 – R1**

Region 1 corresponds to NLS1, a region predicted to confer nuclear localising signal (NLS) propensity. This region is attested to by **Oksenberg and Ahituv** and consists of a conserved tract of positively charged residues (arginine or lysine) within the extreme N-terminus of AUTS2. This region is shared by all AUTS2 family and aAUTS2p homologues. Due to the conserved nature of this region it is likely to confer nuclear localising potential to all AUTS2-related proteins.


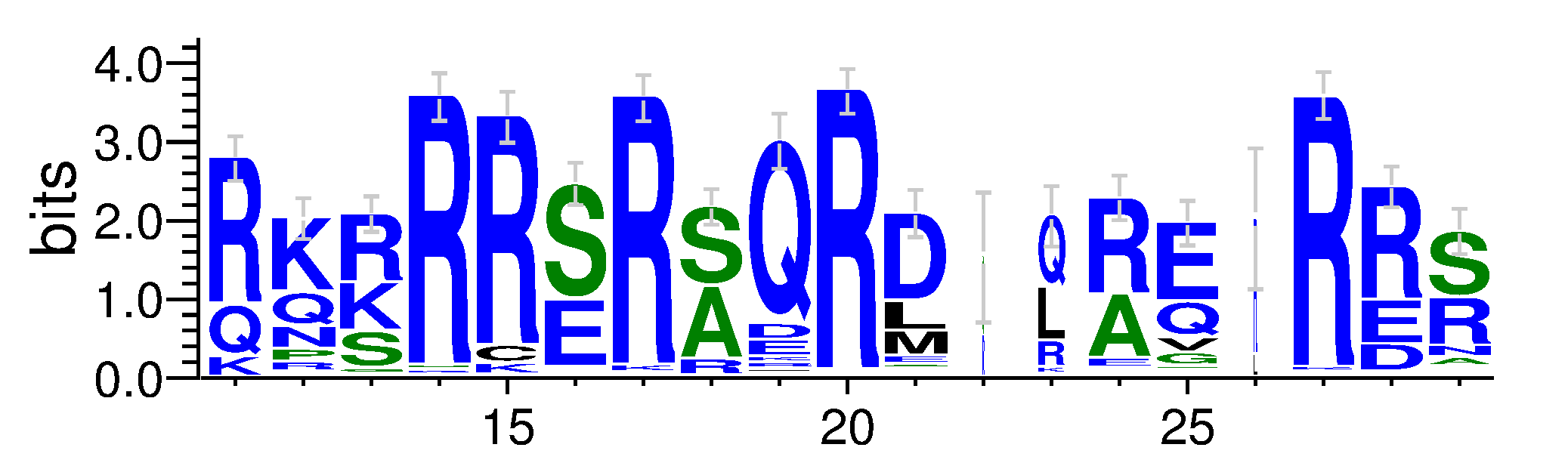


**NLS2 and TayH2 – R2**

Region 2 corresponds to NLS2 and a newly identified hydrophobic region. NLS2 is previously attested to by **Oksenberg and Ahituv** but high conservation within the region immediately downstream has not previously been mentioned. Within Fbrs homologues both elements are separated by a ~30 residue polyglutamate tract, which does not feature within any other related protein. The core of the hydrophobic region, dubbed Tay homology region 2 (TayH2), centres upon a highly conserved motif:

[E][E][-ve][hyd][I][D][G][F][A]

It is possible that this TayH2 region could potentially act as a hydrophobic core for the N-terminal domain (NTD); inferred from a maximum peak in local hydrophobicity, calculated by ProtScale using the Miyazawa hydrophobicity scale [**Gasteiger et al. 2005**; **Miyazawa and Jernigan 1996**]. If TayH2 is a hydrophobic core region this would explain its consistent conservation across all AUTS2-related proteins, as hydrophobic cores are highly conserved structural elements which aid protein folding and stability [**Harris and Pettitt 2016**].


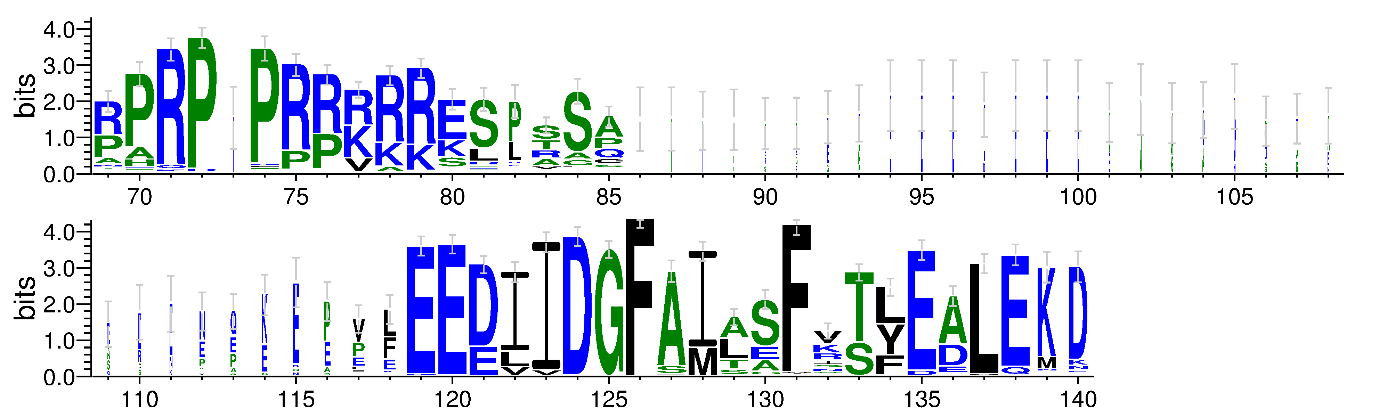


**3-6 Region (36R) – R3**

Region 3 corresponds to a novel region of conservation which comprises the short N-terminal exons of AUTS2 (Exons 4-6). This region, dubbed 3-6 Region (36R; due to its stretching from the C-terminal of Exon 3 to exon 6), contains a highly conserved cysteine-aspartate couplet (C221-D222) located in Exon 5. The 36R is not well conserved in Tay homologues, yet the retention of Exon 5 and 6 homologues within the sequence of Tay may attest to a certain amount of importance. Exon 4 homologues are missing from *Teleost* fish but retained within Coelacanth (*Latimera chalumnae*) and Spotted Gar (*Lepisosteus oculatus*) Auts2 along with *Teleost* Fbrsl1. Fbrs homologues do not contain a homologue for Exon 3 of AUTS2 and therefore are missing the N-terminal end of 36R. Conservation within this region across all AUTS2-related proteins is largely restricted to the region aligned to Exons 5 and 6; no known function has been predicted to reside within this region though S202 is predicted by Scansite to act as a kinase binding.


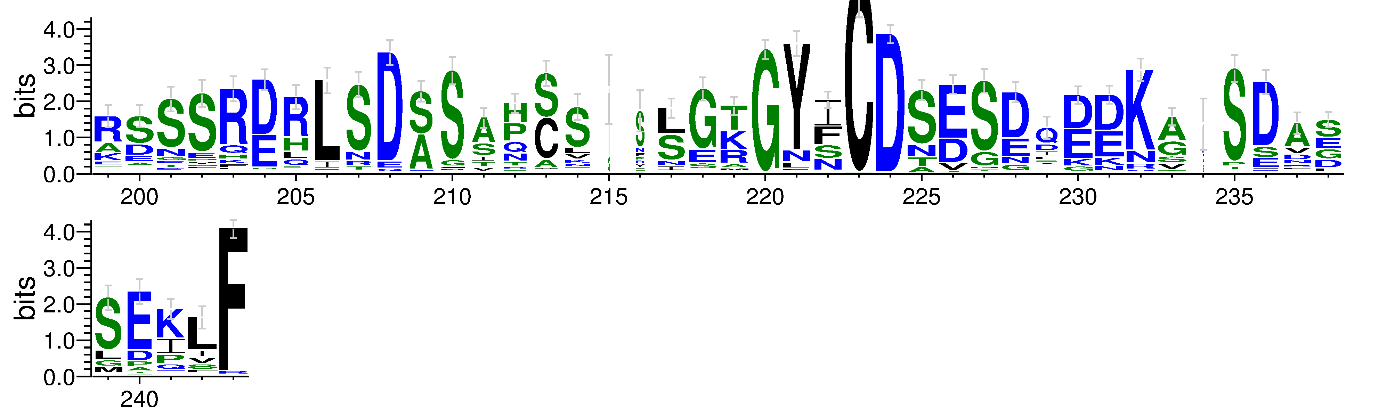


**Tay Domain**

The Tay Domain (TayD) is the most conserved region between AUTS2-related proteins. 7 conserved regions are contained within the TayD spanning from its N- to C-termini. The N-terminal of the TayD is difficult to define as the Serine Rich/Dwarfin Homology region which follows the predicted linker region is predicted to be largely disordered and is unconserved between AUTS2-related proteins though is relatively well conserved between Auts2 homologues. Using the predicted linker region as the domain boundary for the TayD domain structure places the N-terminal at H369, though it may be the case that the structure of the TayD begins to form around E490 within the highly conserved Region 5.

The TayD contains a predicted hydrophobic core region within Exon 14 (Region 8); this region is also displayed as highly conserved and intrinsically ordered which support its predicted role as a hydrophobic core. Exon 10 (Region 7) may also contain a structured core for the TayD as predicted by its low disorder propensity. While the whole of the TayD is shared between all AUT2 homologues, levels of identity vary. Exon 11 is not contained within rodent Fbrsl1 due to a premature stop codon. Exon 12 is commonly spliced out of predicted sequences and features as an ignored exon within an isoform of AUTS2 (AUTS2-002: ENST00000406775.6). Homologues of Exons 12 and 13 do not feature within any aAUTS2p homologues. The HQHQ repeat region is a consistent feature in all AUTS2-related proteins. The WW-binding (PPPY) motif is present and conserved throughout every AUTS2 homologue and a majority of aAUTS2p homologues, excluding Tay homologues, in its degenerate PPxY form. It should be noted that Regions 5-7 are contained within one exon in Tay which may allude to functional partitioning within the TayD.

The C-terminal end of the TayD is equally difficult to define as the possibility exists that the structure of the TayD is not discrete from the C-terminal domain (CTD) though the divergence displayed by the CTD should warrant it status as a variable domain. The most appropriate region to define as a domain boundary is the start of the RERE repeat region which would place the C-terminus of the TayD at H824. The following RERE repeat region is thus classified as part of the CTD but due to its predicted disorder may exist as a discrete flexible element between the TayD and CTD proper.

**ATSS (Alternate Translation Start Site) – R4**

Region 4 corresponds to the alternative translation start site (ATSS) of Auts2 Variant 1 (*Mus musculus*) _[_[_4_](#_ENREF_4)_]_, it should be noted that the methionine (ATG; M459) start codon is conserved throughout all Auts2 and Fbrsl1 homologues and the majority of aAUTS2p homologues, excluding Tay homologues. It should also be noted that no human homologue of Auts2 Variant 1 has yet to be validated though the conservation of M459 does allude to its existence. Region 4 contains a highly conserved polyproline tract and represents the most proximal region of conservation between all AUTS2-related proteins within the TayD.


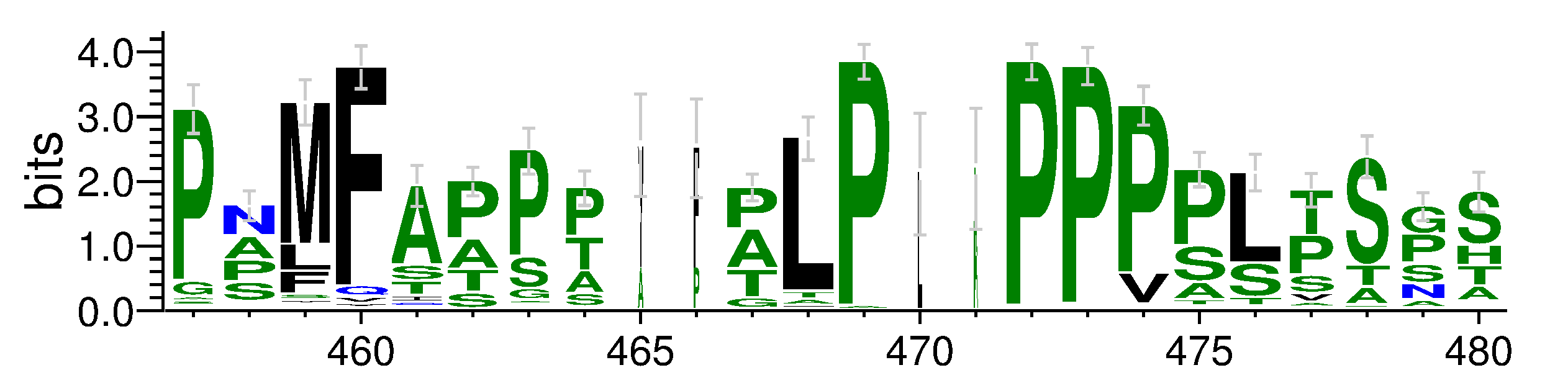


**N-terminal Exon 9 – R5**

Region 5 corresponds to a region within the N-terminus of Exon 9 which is highly conserved throughout all AUTS2-related proteins. This region has not been attested to for any functional importance in any previous study but due to its conservation is likely to contribute to the function of the TayD. The core motif of the region is high in leucine residues and is displayed below:

[-ve][L][L][R]X[E][L]XX[R][F][L]


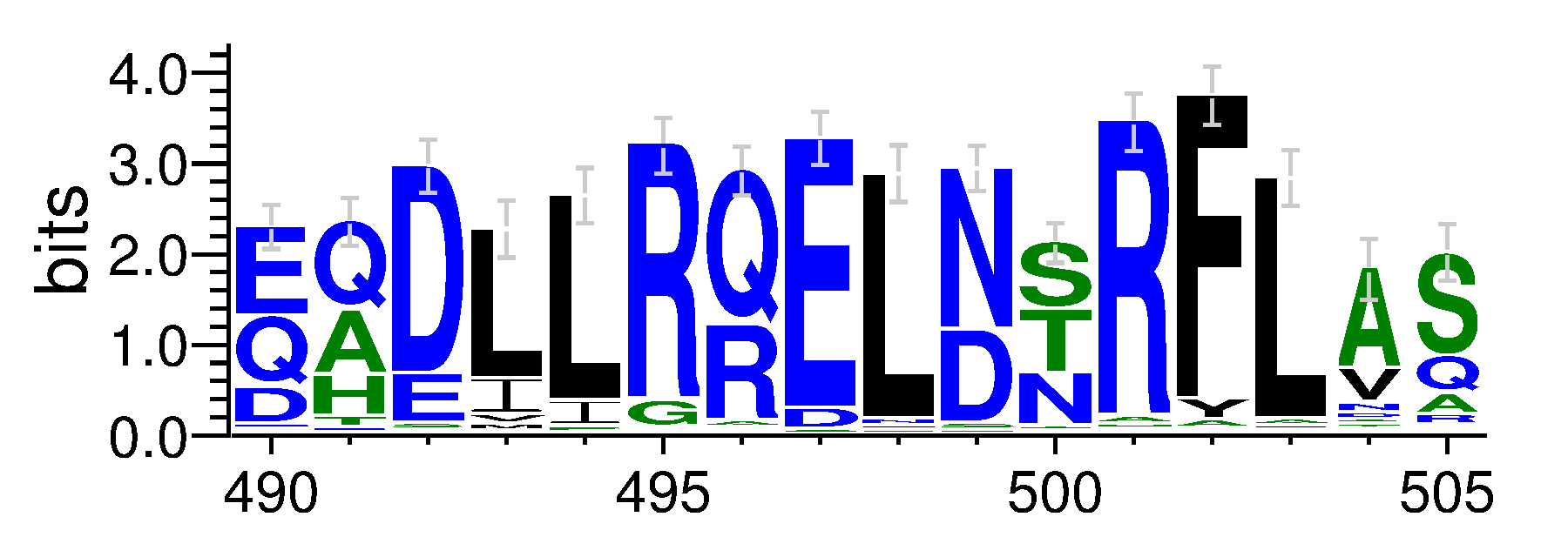


**C-terminal Exon 9 – R6**

Region 6 corresponds to the C-terminal region of Exon 9 (AUTS2) and contains the WW-binding motif and Hexanucleotide repeat element attested to by **Oksenberg and Ahituv** along with part of proline rich region 2 (PR2). The WW-binding motif (PPPY) is conserved throughout all Auts2 homologues and is featured within the majority of aAUTS2p homologues (excluding Tay homologes) in its degenerate PPxY form, still classed as a WW-binding motif harbouring potential for functionality. Immediately downstream of the WW-binding motif is a highly conserved element throughout Auts2, Fbrsl1 and aAUTS2p homologues, with the core motif of:

[L][R]X[E][hyd]

The Hexanucleotide repeat element consists of multiple histidine-glutamine couplets, conserved as an element throughout all AUTS2-related proteins, differing only in the length and substitution of certain glutamine residues for threonine. No known function has been attributed to this region.


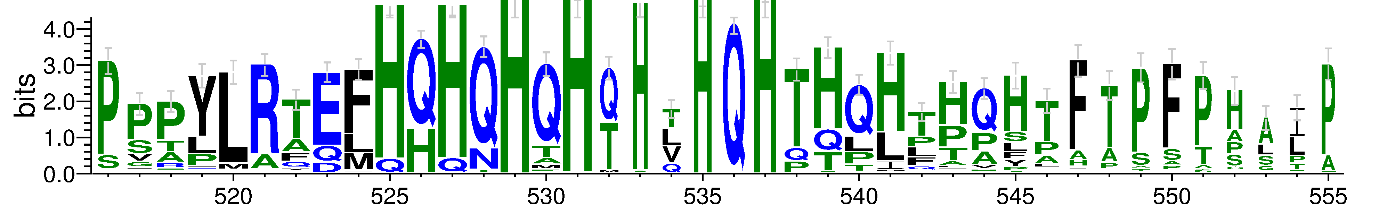


**Exons 10-11 – R7**

Region 7 corresponds to both Exons 10 and 11 of AUTS2. Exon 10 is highly conserved throughout all AUTS2-related sequences. The core motif of Exon 10 is shown below:

[F][-ve][+ve]X[P]X[+ve][hyd][-ve]X[hyd][hyd][R]

Exon 11 is a proline rich region in all AUTS2-related proteins. Exon 11 homologues are not contained within the sequence of rodent Fbrsl1, e.g mouse (*mus musculus*), due to the insertion of a premature stop codon. A highly conserved motif features within the extreme C-terminus of Exon 11 with the consensus:

[F]X[P][K]


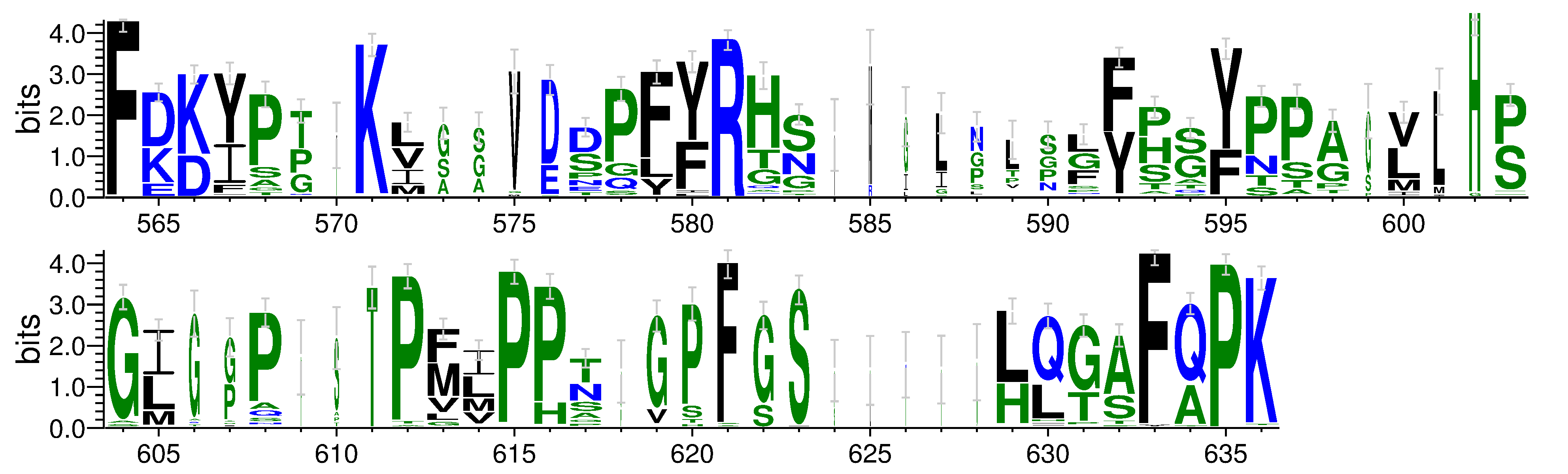


**Exon 14 – R8**

Region 8 corresponds to Exon 14 of AUTS2. Exon 14 is highly conserved across all AUTS2-related proteins and is the central locus of conservation within the Auts2 region (as defined by Pfam). This exon is the most conserved region in aAUTS2p and Tay homologues, with the lowest recorded conservation score being 46.9% identity within the aligned region of *Drosophila elegans* (Fruit fly) aAUTS2p. The consensus motif of the Exon 14 conservation region (R645-K666) is shown below:

[K]X[G][+ve][W]XXX[V][H]X[hyd][A][W]XXXX[Q][Q][K]

The region contains two highly conserved tryptophan residues, W649 and W658 (W658 is substituted for tyrosine in the majority of Fbrs homologues), and is largely composed of hydrophobic residues. From the inclusion of a conserved tryptophan and other hydrophobic residues, it can be inferred that the region likely to be internally stable. A hydrophobicity analysis of AUTS2 displays a strong peak of hydrophobicity visible within the Exon 14 region; potentially meaning that Exon 14 is part of a structural hydrophobic core region within the Tay Domain.


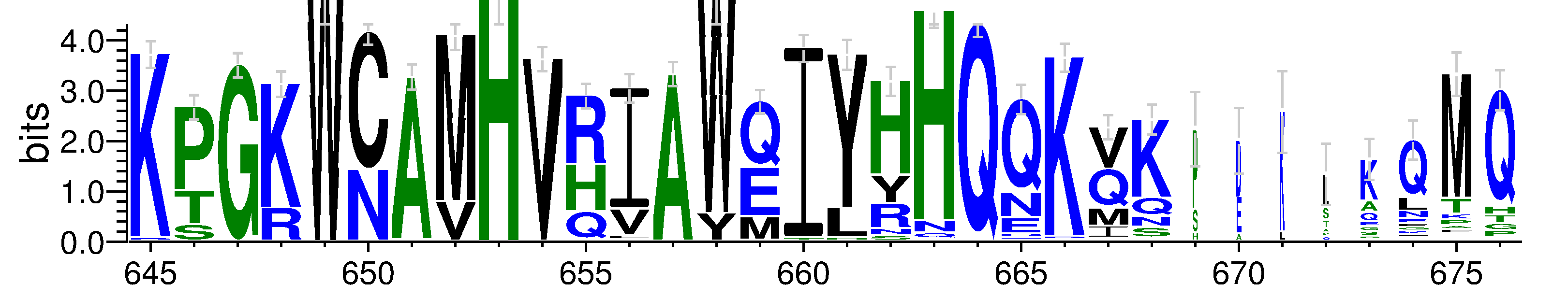


**Exons 16-17 – R10**

Region 9 corresponds to the C-terminus of Exon 16 and the whole of Exon 17. The C-terminus of Exon 16 is proline rich and contains a conserved phenylalanine-leucine couplet (F734-L735) and a histidine-leucine couplet (H740-L741) which are both largely conserved as a hydrophobic couplets through aAUTS2p homologues. Exon 17 is glycine rich within all AUTS2-related proteins and contains a largely conserved [P][F]X[R] motif (P743-R746) and 2 highly conserved phenylalanine residues (F725 and F734). Exon 17 is shorter within in Tay homologues. Both exons display higher levels of identity between AUTS2 family proteins than when compared to aAUTS2p homologues. No functional annotation has previously been made for this region.


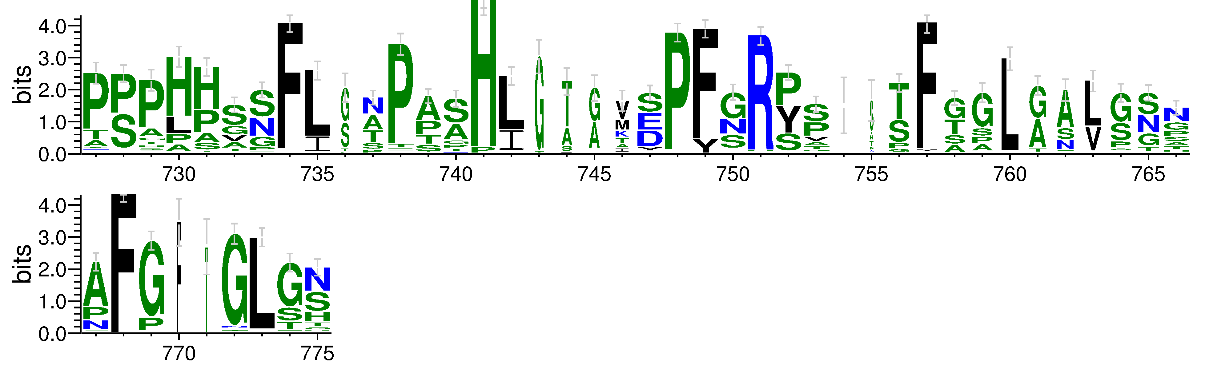


**Exon 18 – R11**

Region 10 corresponds to N-terminal end of AUTS2 Exon 18. Exon 18 contains 2 highly conserved tryptophan residues which feature in the majority of AUTS2-related sequences (W793 and W809; W809 is not contained within the sequence of Frog Fbrs). The intervening region between both tryptophan residues is high in proline residues. No previous functional annotation has been made of Exon 18. The N-terminal end of Exon 18 represents the C-terminus of the Tay domain, preceding a region of alternating positive-negative residues, dubbed the RERE repeat region, within the C-terminus of Exon 18. The distal tryptophan residue (W809) features on a separate chromosome within Tay homologues.


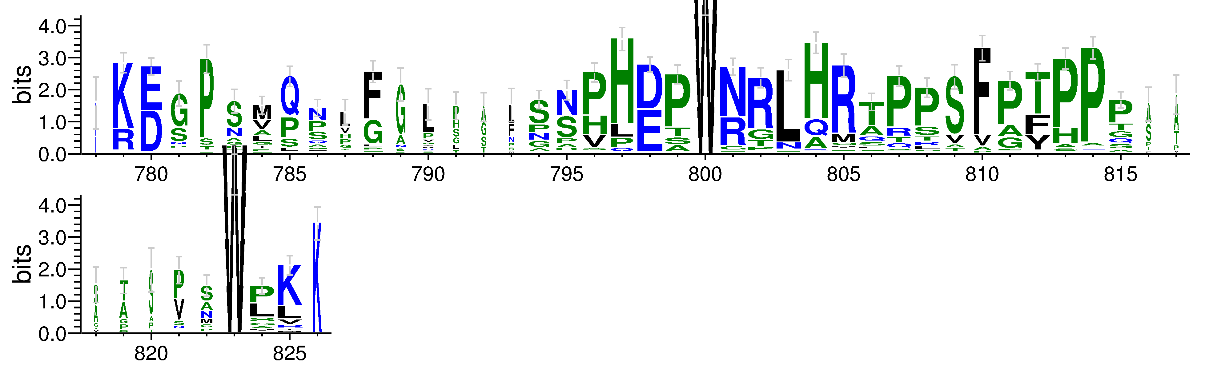


**C-terminal Domain**

The C-terminal Domain (CTD), consisting of the C-terminal of Exon 18 and Exon 19 in AUTS2, is the foremost region of divergence between AUTS2 family sequences. This divergence is most visible when observing the truncation of mammalian CTD homologues in FBRSL1 and FBRS which is contrasted by non-mammalian sequences which retain a length similar to the CTD in AUTS2. Defining the CTD within for this predicted domain structure could either include or exclude the RERE region due to its predicted disorder. The N-terminal end of the CTD would either start at D825 (if RERE is included) or E979 within Region 11 (if RERE is excluded).

The CTD contains a hydrophobic core region common to AUTS2, non-mammalian FBRSL1 and FBRS homologues and aAUTS2p homologues. This core is not as well conserved as TayH2 or Exon 14 but may serve as a structural core for the CTD due to its high level of hydrophobic residues, reasonable conservation and low predicted disorder propensity. Functional evidence surrounding the CTD is sparse but within AUTS2 the region contains two confirmed modifiable serine residues (S1198 and S1233). Using prediction software (NetPhorest) to analyse the modified sites it is predicted that S1198, once phosphorylated, may act as a binding motif for PIN1 (Peptidylprolyl Cis/Trans Isomerase, NIMA-Interacting 1), a post-translational modification enzyme involved in the catalytic regulation of post-phosphorylation conformation in its substrate proteins. PIN1 is reported to regulate the nuclear localisation of multiple proteins related to transcriptional regulation and thus plays an important role in multiple cellular processes [Hanes 2015]. This may implicate S1198 phosphorylation as a key modification, important to the subcellular localisation of AUTS2. S1198 is noted to be phosphorylated through the process of hESC (human embryonic stem cell) differentiation. The trinucleotide (polyhistidine) repeat of AUTS2, immediately downstream of the predicted hydrophobic core, is conserved as an element of all non-fish AUTS2 homologues where fish Auts2 homologues do not contain a polyhistidine tract but do contain a downstream polyalanine tract which does not feature in non-fish Auts2 homologues. Region 11 which caps the RERE region has no known function but exists as a motif in all AUTS2-related proteins.

**Exon 19 Conservation Region – R12**

Region 11 corresponds to a region of Exon 19 in AUTS2 dubbed the Exon 19 conservation region (x19Cons). This region contains a highly conserved motif, conserved across all AUTS2-related proteins. The core motif of this region is shown below:

[Hyd][+ve][Hyd][K][-ve][E]

This region marks the end of the RERE repeat region and precedes a region of conservation potentially acting as a hydrophobic core region for the CTD. Downstream of this region is a large polyalanine tract found in both mammalian Fbrs homologues and Tay homologues.


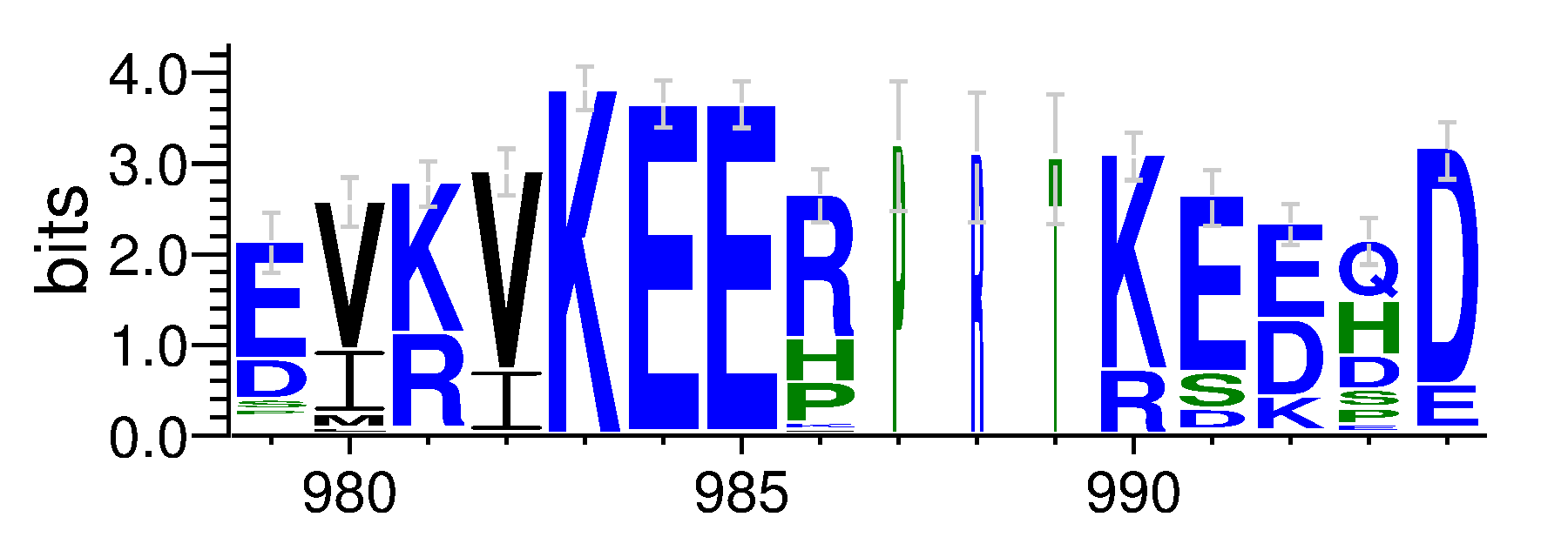


**Characterisation of the N-terminal Domains (NTD)**

The NTD of AUTS2-related proteins display a distinct pattern of localised conservation and divergence (**Figure 5a**). Regions 1-3 overlap with regions of internal conservation, except for Region 1 of both FBRS and Tay (**Figure 5a**; **Supplementary** **Table S6a-e**). The sequence between Regions 1-3 is largely divergent in all AUTS2-related proteins, excluding Tay bridge in which a large region of internal conservation lies upstream of Region 2 *(Region 2c*; **Supplementary** **Table S6e**), likely inferring functionality. A ~30 residue glutamate-rich insertion was identified within Region 2 of mammalian FBRS orthologs (a shorter 10 residue insert is found in reptile FBRS), splitting Region 2 into *Regions* *a* and *b* (**Figure 4**), corresponding to NLS2 and a 19 residue hydrophobic tract respectively; as this disruption of Region 2 is unique to mammalian, and to a lesser extent in reptiles, it may represent a recent sequence insertion within the FBRS sequence. FBRS orthologs lack a region comparable to exon 3 of AUTS2, thus any associated function would be absent in FBRS. *Region d* of AUTS2 aligns to *Region d* of FBRS and is moderately conserved in FBRSL1, but not within any aAUTS2p sequence, therefore may be of functional and/or structural importance to the AUTS2 family proteins.
